# Origin and chromatin remodeling of young X/Y sex chromosomes in catfish with sexual plasticity

**DOI:** 10.1093/nsr/nwac239

**Published:** 2022-10-28

**Authors:** Gaorui Gong, Yang Xiong, Shijun Xiao, Xi-Yin Li, Peipei Huang, Qian Liao, Qingqing Han, Qiaohong Lin, Cheng Dan, Li Zhou, Fan Ren, Qi Zhou, Jian-Fang Gui, Jie Mei

**Affiliations:** Hubei Hongshan Laboratory, College of Fisheries, Huazhong Agricultural University, Wuhan 430070, China; Hubei Hongshan Laboratory, College of Fisheries, Huazhong Agricultural University, Wuhan 430070, China; Jiaxing Key Laboratory for New Germplasm Breeding of Economic Mycology, Jiaxing 314000, China; State Key Laboratory of Freshwater Ecology and Biotechnology, Hubei Hongshan Laboratory, Institute of Hydrobiology, Chinese Academy of Sciences, University of the Chinese Academy of Sciences, Wuhan 430072, China; Hubei Hongshan Laboratory, College of Fisheries, Huazhong Agricultural University, Wuhan 430070, China; School of Animal Science and Nutritional Engineering, Wuhan Polytechnic University, Wuhan 430023, China; Hubei Hongshan Laboratory, College of Fisheries, Huazhong Agricultural University, Wuhan 430070, China; Hubei Hongshan Laboratory, College of Fisheries, Huazhong Agricultural University, Wuhan 430070, China; Hubei Hongshan Laboratory, College of Fisheries, Huazhong Agricultural University, Wuhan 430070, China; State Key Laboratory of Freshwater Ecology and Biotechnology, Hubei Hongshan Laboratory, Institute of Hydrobiology, Chinese Academy of Sciences, University of the Chinese Academy of Sciences, Wuhan 430072, China; State Key Laboratory of Freshwater Ecology and Biotechnology, Hubei Hongshan Laboratory, Institute of Hydrobiology, Chinese Academy of Sciences, University of the Chinese Academy of Sciences, Wuhan 430072, China; State Key Laboratory of Freshwater Ecology and Biotechnology, Hubei Hongshan Laboratory, Institute of Hydrobiology, Chinese Academy of Sciences, University of the Chinese Academy of Sciences, Wuhan 430072, China; Hubei Hongshan Laboratory, College of Fisheries, Huazhong Agricultural University, Wuhan 430070, China; MOE Laboratory of Biosystems Homeostasis & Protection, Life Sciences Institute, Zhejiang University, Hangzhou 310058, China; Hubei Hongshan Laboratory, College of Fisheries, Huazhong Agricultural University, Wuhan 430070, China; State Key Laboratory of Freshwater Ecology and Biotechnology, Hubei Hongshan Laboratory, Institute of Hydrobiology, Chinese Academy of Sciences, University of the Chinese Academy of Sciences, Wuhan 430072, China; Hubei Hongshan Laboratory, College of Fisheries, Huazhong Agricultural University, Wuhan 430070, China

**Keywords:** Y chromosome, sex-chromosome evolution, chromosome fusion, chromatin organization, sexual reversal

## Abstract

Assembly of a complete Y chromosome is a significant challenge in animals with an XX/XY sex-determination system. Recently, we created YY-supermale yellow catfish by crossing XY males with sex-reversed XY females, providing a valuable model for Y-chromosome assembly and evolution. Here, we assembled highly homomorphic Y and X chromosomes by sequencing genomes of the YY supermale and XX female in yellow catfish, revealing their nucleotide divergences with only less than 1% and with the same gene compositions. The sex-determining region (SDR) was identified to locate within a physical distance of 0.3 Mb by F_ST_ scanning. Strikingly, the incipient sex chromosomes were revealed to originate via autosome–autosome fusion and were characterized by a highly rearranged region with an SDR downstream of the fusion site. We found that the Y chromosome was at a very early stage of differentiation, as no clear evidence of evolutionary strata and classical structure features of recombination suppression for a rather late stage of Y-chromosome evolution were observed. Significantly, a number of sex-antagonistic mutations and the accumulation of repetitive elements were discovered in the SDR, which might be the main driver of the initial establishment of recombination suppression between young X and Y chromosomes. Moreover, distinct three-dimensional chromatin organizations of the Y and X chromosomes were identified in the YY supermales and XX females, as the X chromosome exhibited denser chromatin structure than the Y chromosome, while they respectively have significantly spatial interactions with female- and male-related genes compared with other autosomes. The chromatin configuration of the sex chromosomes as well as the nucleus spatial organization of the XX neomale were remodeled after sex reversal and similar to those in YY supermales, and a male-specific loop containing the SDR was found in the open chromatin region. Our results elucidate the origin of young sex chromosomes and the chromatin remodeling configuration in the catfish sexual plasticity.

## INTRODUCTION

The XX/XY chromosome system is the most common sex-determination system in vertebrates. In mammals, sex is usually determined by the major sex-determining region (SDR) on the Y chromosome [1]. Complete and high-quality Y-chromosome sequences are essential for studying the evolution and mechanism of sex determination [2]. Among most sequencing projects, individuals of homogametic sex (XX females) are usually chosen for genome sequencing and assembly, whereas whole Y-chromosome sequences are assembled mainly in a handful of XY-male mammals through a time- and labor-intensive clone-by-clone approach [3–7], as well as in YY-supermale fishes generated by artificial sex-reversal technology [8,9], and in XY-male fish by telomere-to-telomere assembly [10]. It is widely accepted that sex chromosomes evolve from a pair of autosomes, one of which acquires a sex-determining locus. Next, the progressive evolutions of recombination suppression though chromosome rearrangements lead to the formation of heteromorphic X and Y chromosomes [11–14]. The prevailing models of sex-chromosome origin and evolution are mainly based on the stable and highly differentiated Y chromosomes in mammals. However, sex-chromosome evolution and the recombination suppression mechanism are more complex than previously expected and a broader understanding of vertebrate sex chromosomes is hindered by the limited number and no availability of high-quality Y-chromosome sequences [15].

In contrast to the heteromorphic sex chromosomes of mammals, homomorphic sex chromosomes with poor differentiation are observed in many lower vertebrates, including most fish species, which provide a good model to study the initial stages of sex-chromosome evolution [16,17]. During the process of sex-chromosome evolution, multiple sex-chromosome constitutions have been frequently observed in fishes and reptiles, resulting from the fusion of sex chromosomes and autosomes, as whole-chromosome fusions are more frequent on sex chromosomes than in autosomes [18–20]. Recently, it was proposed that autosome–autosome fusions occurred before the recruitment of the fused autosomes as sex chromosomes [21]. However, there is no available model to investigate the evolutionary mechanism of sex-chromosome formation via autosome–autosome fusion.

Sex determination in fish and amphibians is a plastic process that can be modulated by both genetic and environmental factors [22,23]. The most common form of environmental sex determination (ESD) in fish and amphibians is temperature-dependent sex determination (TSD), which is controlled by epigenetic modifications, such as genomic DNA methylation [24] and histone modification [25,26]. It is widely recognized that epigenetic modifications can modulate chromatin architecture and dynamics, which play critical roles in gene-expression regulation, cell differentiation and developmental processes [27,28]. Temperature-sensitive and temperature-insensitive populations exist simultaneously in some fish and amphibian species, and transitions between genetic sex determination (GSD) and TSD have been reported in gibel carp, yellow catfish, Nile tilapia and bearded dragon [29–34] and Y-autosome fusion was revealed to generate neo-Y chromosomes in fish and amphibians [18,35]. However, it remains unclear whether the chromatin architecture of poorly differentiated Y chromosomes contributes to sex determination and male-biased gene expression.

Recently, YY-genotype male yellow catfish, channel catfish and southern catfish have been generated via the integration of hormonally induced sex-reversal technology and sex-linked marker identification [8,9,36,37], providing a unique research model for studying the structure and evolution of sex chromosomes in catfishes. Sexually reversed XX-neomale yellow catfish were artificially produced by using an aromatase inhibitor, letrozole, and artificially induced sex reversal leads to a transition from GSD to TSD in yellow catfish [30]. In this study, we assembled and compared X- and Y-chromosome sequences of yellow catfish by sequencing the genomes of XX females and YY supermales to explore the origin and evolution of homomorphic X and Y chromosomes with an XX/XY sex-determining system. Strikingly, sex chromosomes in yellow catfish were revealed to originate from autosome–autosome fusion. We further compared the chromatin architectures of the X and Y chromosomes in XX-female, XX-neomale and YY-supermale yellow catfish and found the potential roles of the 3D chromatin architecture in fish sex determination and sexual plasticity.

## RESULTS

### Identification of sex chromosomes by bacterial artificial chromosome–fluorescence *in situ* hybridization (BAC–FISH)

Sex-chromosome-linked markers and BAC clones containing sex-chromosome-linked markers have been identified in previous work [36]. To identify the sex chromosomes of yellow catfish, we performed FISH on three different genotypes of yellow catfish (XX female, XY male and YY supermale) with BAC DNA as a probe. As shown in Supplementary Fig. 1A, two signal loci were observed on a pair of homologous chromosomes in metaphase cells of XY males. Based on the hybridization signals of sex-chromosome-linked BAC localization, we identified the sex chromosomes of yellow catfish. A similar localization and the same number of hybridization signals were observed in the metaphase cells of XX females and YY supermales (XX: Supplementary Fig. 1B; YY: Supplementary Fig. 1C).

The karyotypic analysis of somatic cells indicates that the chromosome number of yellow catfish is 2*n* = 52. According to the identified sex-specific markers and chromosome-level assembly of the yellow-catfish genome [36,38], Chromosome 2 is the sex chromosome and has the second largest size among all chromosome pairs (Supplementary Fig. 1D). We infer that the sex chromosomes are a pair of submetacentric chromosomes based on their morphological features along with the hybridization signals of sex-linked markers on their two long arms (Supplementary Fig. 1E). However, in XY-genotype metaphases, the BAC DNA probe could not directly distinguish the X and Y chromosomes via the FISH method. The highly homomorphic structure of this sex-chromosome pair may be the reason why we could not distinguish them using traditional cytogenetic methods. This also suggests that this sex-chromosome pair is still in the initial stage of evolution.

### Accurate genome assembly of YY supermale and XX female

Whole-genome sequencing and assembly were performed on an YY supermale and XX female to explore their genomic differences in yellow catfish. A YY supermale was chosen for genome sequencing using a combination of several technologies, including single-molecule real-time sequencing (PacBio RS II), paired-end sequencing (Illumina HiSeq 2000) and chromatin conformation capture (Hi-C). Finally, these sequences were assembled into 1590 contigs, with a contig N50 of 2.95 Mb and a scaffold N50 of 27.04 Mb. The total assembled genome sequence of YY supermale was 710 Mb, while the total length of the anchored contigs was ∼706 Mb. A total of 94.7% of the yellow catfish genes completely matched the Benchmarking Universal Single-Copy Orthologs (BUSCO) set (Supplementary Table 1). In addition, the XX genome [38] was reassembled and improved according to the same assembly methods as for the YY genome. The XX genome sequences were finally assembled into 1335 contigs, with a contig N50 of 3.16 Mb and a scaffold N50 of 27.16 Mb. The total assembled genome sequence of the XX yellow catfish was 710 Mb (Supplementary Table 1). A total of 95.0% of the XX yellow-catfish genes completely matched the BUSCO set and the assembly metrics showed considerable improvement relative to the previous assembly [38]. To further evaluate assembly completeness, the genomic sequences of XX and YY yellow catfish were directly aligned with LAST and extremely high sequence similarity was found (Supplementary Fig. 2).

### Subtle divergence of repetitive sequence subfamilies between X and Y chromosomes

**Figure 1.**
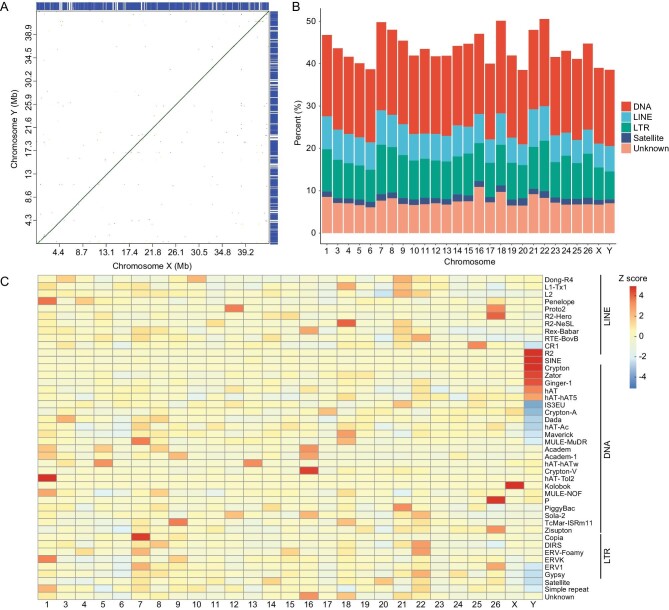
Sequence comparisons between the X and Y chromosomes. (A) Comparison of X- and Y-chromosome sequences. The *y*-axis represents the Y chromosome and the *x*-axis represents the X chromosome. (B) The X- and Y-chromosome genes (blue) share the same content and gene order. The bar chart shows the proportions of several repeat families on each chromosome. (C) The abundance of each repeat subfamily (right side) was normalized according to the Z score for each chromosome.

We next scrutinized the sequence differences between the X and Y chromosomes to reveal the potential sex-determination genes. The assembled X chromosome was 43.5 Mb in length and composed of 39 contigs, while the assembled Y chromosome was 43.2 Mb and composed 48 contigs (Supplementary Table 2). The alignment of the X and Y chromosomes showed >99% sequence identity. In addition, we found highly similar distributions of the GC contents and repeat contents of the X and Y chromosomes (Supplementary Figs 3 and 4). A total of 1427 genes were annotated on both the assembled Y chromosome and X chromosome, and the gene content was completely shared between the X and Y chromosomes (Fig. 1A). In an attempt to discover whether different evolutionary strata existed on the yellow-catfish sex chromosomes, as observed in some model fish species, including three-spined stickleback [39] and spotted knifejaw [40], we estimated non-synonymous site divergence (Ka), synonymous site divergence (Ks) and Ka/Ks between the X and Y chromosomes but did not find any regions with significant differences in these parameters (Supplementary Figs 5–7).

We further analysed the repetitive sequence divergence between the X and Y chromosomes. A total of 39.88% of the X chromosome and 39.48% of the Y chromosome consisted of repeat sequences, which was comparable to the rates among autosomes (39.37%–51.38%). The repetitive elements identified on the X and Y chromosomes were mostly DNA transposons (17.88% and 18.02%, respectively), long terminal repeat (LTR) retrotransposons (LTRs, 7.62% and 6.59%, respectively) and long interspersed elements (LINEs, 5.64% and 5.97%, respectively) (Fig. 1B). However, the classification of repetitive DNA sequences into subfamilies revealed that several subfamilies of DNA transposons (Crypton, Zator, Ginger-1, hAT and hAT-hAT5), LINE/R2 elements and short interspersed nuclear elements (SINEs) have specifically accumulated on the Y chromosome relative to the rest of the genome. In contrast, some subfamilies of DNA transposons (IS3EU, Crypton-A, Dada, hAT-Ac and Maverick) and LTRs (ERV1 and Gypsy) showed a relatively lower content on the Y chromosome (Fig. 1C). Additionally, we found obvious differences in the distributions of DNA/hAT, DNA/Zator, DNA/Crypton-A, SINE and LINE/CR1 elements between the X and Y chromosomes (Supplementary Fig. 8). The analysis of the sequence divergence of individual families from the inferred consensus sequences revealed recent changes on the X and Y chromosomes. Several DNA transposon subfamilies (Crypton/A, Kolobok and Merlin) showed higher divergence on the X chromosome, while higher divergence of other DNA transposons (Maverick, IS3EU and hAT/Blackjack) and LTRs (ERVK and Gypsy) was observed on the Y chromosome (Supplementary Fig. 9). Taken together, these results indicate that subtle divergence of repetitive sequence subfamilies have occurred on the X and Y chromosomes, suggesting that they are still in a very early stage of sex-chromosome differentiation.

### Identification of the SDR

**Figure 2.**
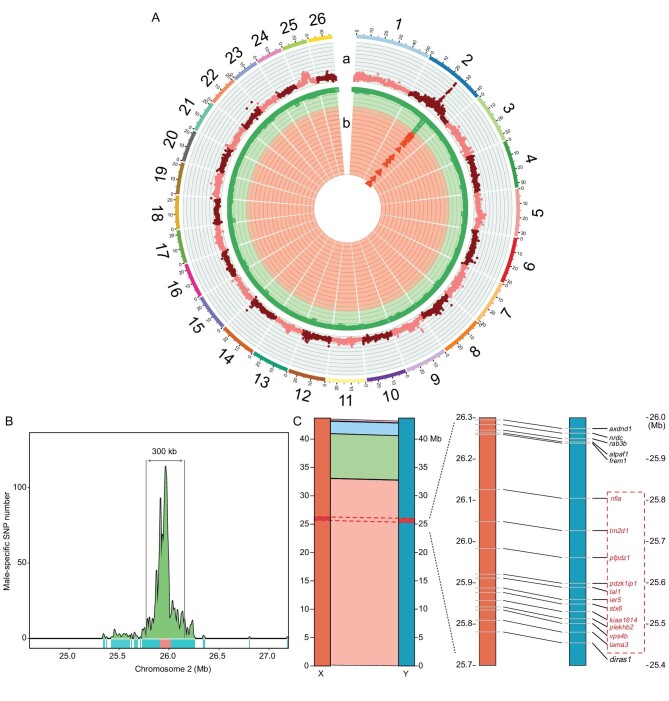
Characterization of the sex-determination region and sex chromosome in yellow catfish. (A) Circular plot showing the whole-genome analysis of F_ST_ and the male-specific SNP distribution. (a) The outer track shows the genome-wide F_ST_ statistics calculated using a 10-kb sliding window with a step of 5 kb. (b) The inner track shows the male-specific SNP distribution calculated using a 20-kb sliding window with a step of 5 kb. (B) An ∼300-kb region significantly enriched with male-specific SNPs was identified on Chromosome 2. The region where the BAC sequence is located is highlighted in red. (C) Homology between the X and Y chromosomes. The left panel shows the locally collinear blocks (LCBs) shared by the X and Y chromosomes. Each LCB is color-coded and represents a syntenic region. The SDR is highlighted in red. The right panel shows the same gene order around the SDR. The genes in the SDR are highlighted in red.

Pooled sequencing reads from the genomic DNA of 20 XX females and 20 YY supermales were mapped to the XX genome to further characterize genomic regions enriched for sex-biased signals (Supplementary Table 3). We obtained a large number of variants (3 476 961 single-nucleotide polymorphisms [SNPs] and 1 359 630 indels) and searched for the variants that were not only fixed in the XX-female pool but were also homozygous mutants in the YY-supermale pool. As a result, 3001 male-specific SNPs and 2285 male-specific indels were identified across the genome, 1809 and 714 of which were concentrated within an ∼400-kb region on Chromosome 2, and this region showed an increase in the fixation index (F_ST_) relative to other regions of the sex chromosomes or autosomes (Fig. 2A). To further confirm these sex-specific variants, we collected additional 19 XX females and 19 YY supermales from another two families and performed re-sequencing (Supplementary Table 3). After taking the intersection of the male-specific variants between these two batches of samples, a number of sex-antagonistic mutations including 714 male-specific SNPs and 248 male-specific indels were identified, all of which were located on Chromosome 2. In addition, the vast majority of these SNPs (617 of 714) and indels (199 of 248) were within a physical distance of 0.3 Mb (Fig. 2B and Supplementary Fig. 10). The BAC DNA sequence containing sex-chromosome-linked markers was consistently located in this region, suggesting that it should be the SDR of yellow catfish (Fig. 2B). We identified four synteny blocks between the X and Y chromosomes, which covered the entire sex chromosomes. The SDRs of the X and Y chromosomes were located in the largest synteny block and shared the same gene contents in the same order, without any chromosome inversions or translocations (Fig. 2C).

The 487 male-specific SNPs in this region were specifically located in 11 genes, namely *nfia, tm2d1, pfpdz1, pdzk1ip1, tal1, ier5, stx6, kiaa1614, plekhb2, vps4b* and *lama3*, with 375 (77.6%) of these male-specific SNPs being located in *pfpdz1*. Furthermore, only five genes had Y-specific SNPs on the exons, including *lama3, vps4b, kiaa1614, tal1* and *pfpdz1* (Supplementary Table 4). During the period of sex determination and differentiation in yellow catfish, the mRNA expressions of *nfia, pfpdz1, tal1, stx6* and *kiaa1614* in the testis of XY males were higher than in the ovary of XX females (Supplementary Fig. 11), whereas the expression of *pdzk1ip1* was very low and could not be detected. By analysing the distribution of transposable elements on *pfpdz1X* and *pfpdz1Y*, three Y-specific DNA transposons were identified, including two DNA/CACTA and one DNA/hAT transposons (Supplementary Fig. 12A). For the retrotransposons, there are a number of Y-specific LTRs and one Y-specific LINE (Supplementary Fig. 12B).

### Origin and evolution of the young sex chromosomes by autosome–autosome fusion

**Figure 3.**
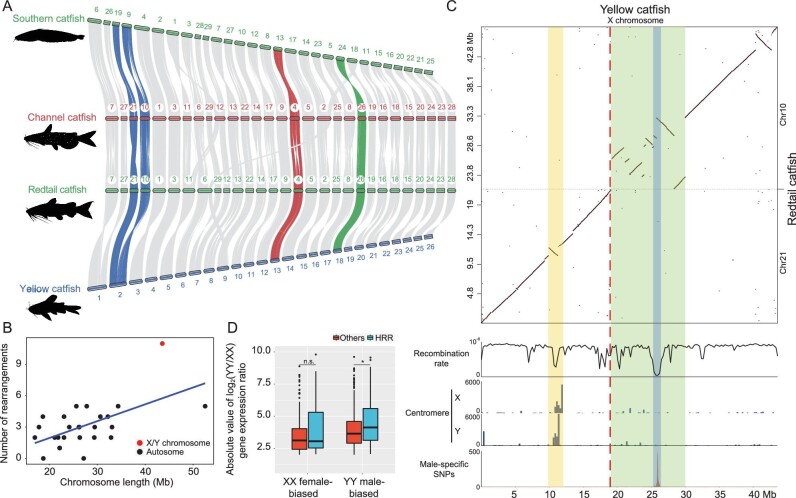
Origin and evolution of yellow catfish sex chromosomes. (A) Genome-scale synteny analysis of yellow catfish (*Pelteobagrus fulvidraco*), redtail catfish (*Mystus wyckioides*), channel catfish (*Ictalurus punctatus*) and southern catfish (*Silurus meridionalis*) by using MCscan. Chromosomes 2, 26, 4 and 24 are the sex chromosomes of yellow catfish, redtail catfish, channel catfish and southern catfish, respectively. The one-to-one correspondence of homologous regions between the sex chromosomes of each species and chromosomes of other species is highlighted in different colors. (B) Dot plot showing the correlation between the overall redtail catfish versus yellow catfish synteny block rearrangement number per chromosome vs the size of the chromosome. Each dot represents a chromosome. (C) Rearrangements between the X chromosome of yellow catfish and Chromosomes 10 and 21 of redtail catfish. In the top panel, the dot plot shows the sequence alignment between the X chromosome of yellow catfish and Chromosomes 10 and 21 of redtail catfish. The second panel shows the recombination rate (c/bp) estimated with variable window sizes based on the number of available variants. The centromere panel shows the putative centromere repeat sequence distribution along the X and Y chromosomes. The last panel shows the distribution of male-specific SNPs along the X chromosome. The putative centromere region is highlighted in yellow and the highly rearranged region (HRR) is highlighted in green. The SDR is shaded in blue and the fusion site is divided with a red dotted line. (D) YY male-biased genes in the HRR region show a higher expression ratio than in other regions of the sex chromosome.

Recently, chromosome-level genome assemblies and sex-chromosome-linked markers of channel catfish (*Ictalurus punctatus*), southern catfish (*Silurus meridionalis*) and redtail catfish (*Mystus wyckioides*) have been reported [9,41–44]. Along with the chromosomal assemblies of the XX and YY yellow-catfish genomes, we were motivated to explore origin and evolution of the sex chromosomes. All four of these species belong to Siluriformes, among which the southern catfish belongs to the family Ictaluridae, channel catfish belongs to the family Siluridae and redtail catfish and yellow catfish both belong to the family Bagridae. Based on protein sequence similarity, chromosomal collinear synteny among southern catfish, redtail catfish, channel catfish and yellow catfish was evaluated (Fig. 3A). All three fish species other than yellow catfish have 29 pairs of chromosomes (2*n* = 58) and the chromosomes of these three species are highly syntenic to each other, indicating relatively conserved karyotypes in Siluriformes. The sex chromosome of channel catfish (Chr4) shares homology with Chr13 of yellow catfish, while the sex chromosomes of southern catfish (Chr24) and redtail catfish (Chr26) are homologous to Chr18 of yellow catfish. Notably, the sex chromosomes of yellow catfish, redtail catfish and channel catfish share no homology, while southern catfish shows the same sex chromosome as redtail catfish (Fig. 3A). Due to the closer relationship between yellow catfish and redtail catfish than the other species, we performed interchromosomal rearrangement analysis between them. We observed several chromosome fissions or fusions between yellow catfish and redtail catfish, including large interchromosomal rearrangements on four chromosomes, namely Chr1, Chr2, Chr7 and Chr9, of yellow catfish (Supplementary Fig. 13). Surprisingly, we found that the sex chromosomes of yellow catfish were probably derived from the fusion of two autosomes that did not undergo interchromosomal rearrangements in the other three species (Supplementary Fig. 14). Synteny analysis between yellow catfish and electric eel (belonging to Gymnotiformes, which is thought to be the sister group to Siluriformes [45,46]) further confirmed the existence of a fusion site at ∼19 Mb in the X/Y chromosomes of yellow catfish (Supplementary Fig. 15).

In addition, we found that there were many more intrachromosomal rearrangements (inversions and translocations) in the yellow-catfish sex chromosomes than in any of the autosomes by sequence alignment with redtail catfish (Fig. 3B). Therefore, we examined the sequence synteny between the X chromosome of yellow catfish and the two corresponding autosomes of redtail catfish. The vast majority of the observed intrachromosomal rearrangements occurred in a highly rearranged region (HRR) in the downstream half of the fusion site and the SDR was located near the middle of the HRR (Fig. 3C). In addition, we observed that the recombination rate of the yellow catfish sex chromosomes was low near the fusion site and close to 0 in the SDR, consistently with the theory of sex-chromosome evolution[47,48]. Additionally, we annotated the putative centromeric region by searching the most abundant satellite sequences and found that this region consisted of an inversion and showed a low recombination rate (Fig. 3C). The sequence alignments between the sex chromosomes of yellow catfish and the corresponding autosomes of channel catfish and southern catfish revealed the same results (Supplementary Fig. 16). Due to the high ratio of translocations and inversions, we speculated that the HRR may have the potential to form a new stratum. By examining transcriptomic expression profiles in gonads of XX-female and YY-supermale yellow catfish, 363 male-biased and 235 female-biased genes were identified on the sex chromosomes, of which 93 male-biased and 78 female-biased genes were located in the HRR. The male-biased genes in the HRR showed significantly higher upregulation compared to other regions of sex chromosomes (*P-*value = 0.02365, Wilcoxon test), while the female-biased genes did not (*P*-value = 0.3068, Wilcoxon test) (Fig. 3D).

### Diverse 3D chromatin architecture on X and Y chromosomes

**Figure 4.**
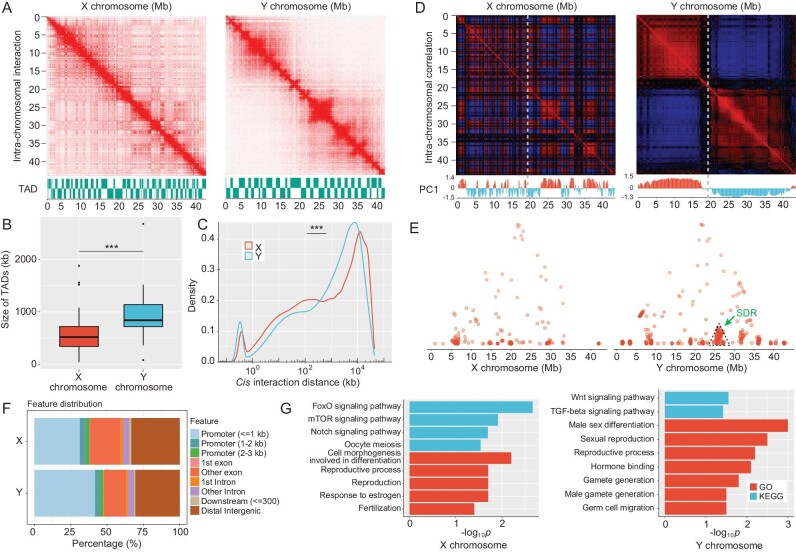
Comparison of the chromatin structure of sex chromosomes in XX females and YY supermales. (A) Hi-C interaction heat maps (50-kb resolution) of the X and Y chromosomes. Identified TADs are shown at the bottom. (B) Correlation heat maps (100-kb resolution) of the X and Y chromosomes. Under the heat map, we show the PC1 score of the Hi-C interaction matrix. (B) The sizes of X-chromosome TADs (*n* = 71) and Y-chromosome TADs (*n* = 42). Wilcoxon test, ***P < 0.001. (C) Comparison of the *cis*-interaction distribution between the X and Y chromosomes. All *cis* interactions of the X or Y chromosomes were used to plot the density plots. Wilcoxon test, ***P < 2.2 × 10^−16^. (D) Correlation heat maps (100-kb resolution) of the X and Y chromosomes. Under the heat map, we show the PC1 score of the Hi-C interaction matrix. (E) Dot plot of significant *cis* interactions of X and Y chromosomes. Each dot represents a significant *cis* interaction of the X and Y chromosomes. The green arrow indicates the SDR of the Y chromosome. (F) Regions that have significant *trans* interactions with X and Y chromosomes mapping to promoters, exons, introns and intergenic regions. (G) GO and KEGG enrichment of annotated genes showing significant *trans* interactions with the X and Y chromosomes. GO terms and KEGG pathways involved in sex determination, gonadal development and gametogenesis are shown.

Although the X and Y chromosomes of yellow catfish showed high sequence identity, their genomic DNA could be packaged into different 3D chromatin architectures because of different repeat contents or transcription factor binding sites shaping the chromatin architecture. To test this hypothesis, we inferred genome-wide chromatin interaction frequencies by performing Hi-C experiments in gonad cells of XX females and YY supermales and obtained ∼463 million and ∼459 million read pairs, respectively. After filtering, a total of 225 million and 205 million valid read pairs were produced in the XX-female and YY-supermale gonad cells, respectively. The generated contact maps showed obvious differences, with a Pearson correlation of 0.88 (Fig. 4A and Supplementary Fig. 17). Seventy-one and 42 topologically associated domains (TADs) were identified on the X chromosome and Y chromosome, respectively. We found that only one TAD shared the same boundaries on the X and Y chromosomes. The TADs on the Y chromosome were larger than those on the X chromosome (*P*-value = 4.884 × 10^−7^, Wilcoxon test) (Fig. 4B). Compared to the Y chromosome, the X chromosome exhibited a higher proportion of short-range (0.3–160 kb) *cis* interactions than long-range (>10 Mb) *cis* interactions, while the Y chromosome presented more mid-range (160 kb–10 Mb) *cis* interactions, indicating that the X chromosome structure is relatively denser (Fig. 4C). The conversion of Hi-C contact frequencies into Pearson correlation coefficients further showed obvious differences between the X and Y chromosomes (Fig. 4D).

Compared to the segregated A and B compartments of chromosome X, the chromatin compartments of chromosome Y were divided into one large A compartment (Y1A, 0–17.85 Mb) and one large B compartment (Y1B, 19.60–40.65 Mb), with an expectedly greater number of short-range (<1.5 Mb) *cis* interactions in Y1B than in Y1A (*P-*value < 2.2 × 10^−16^, Wilcoxon test) (Supplementary Fig. 18). Moreover, we observed that the A compartment presented more *trans* interactions with autosomes than the B compartment on both the X (*P*-value < 2.2 × 10^−16^, Wilcoxon test) and Y (*P*-value = 6.713 × 10^−4^, Wilcoxon test) chromosomes (Supplementary Fig. 19). Additionally, we found significant *cis* interactions on the Y chromosome that were obviously enriched near the SDR but were not observed on the X chromosome (Fig. 4E). To explore whether sex chromosomes maintain the stability of sex characteristics by interacting with autosomes, we identified 2389 and 3118 significant *trans* interactions between autosomes and the X or Y chromosome, respectively. A total of 47.5% of the significant Y-chromosome *trans* interactions were mapped to promoter regions, which was higher than the percentage on the X chromosome (38%). In contrast, the X chromosome showed a higher proportion (21%) of significant *trans* interactions that mapped to exon regions than the Y chromosome (15.6%) (Fig. 4F). Finally, the significant *trans* interactions of the X chromosome and Y chromosome were annotated to 1343 and 1554 genes, respectively. Gene Ontology (GO) and Kyoto Encyclopedia of Genes and Genomes (KEGG) pathway analyses revealed that the genes that presented significant interactions with the X chromosome were functionally enriched in reproduction, the response to estrogen, fertilization, FoxO signaling, mTOR signaling, Notch signaling and oocyte meiosis. Furthermore, the genes that presented significant interactions with the Y chromosome were involved in male-sex differentiation, reproduction, hormone binding, gamete generation, germ-cell migration and the Wnt signaling and TGF-beta signaling pathways (Fig. 4G).

### Three-dimensional chromatin reorganization coordinating with gene-expression regulation in response to sex reversal

**Figure 5.**
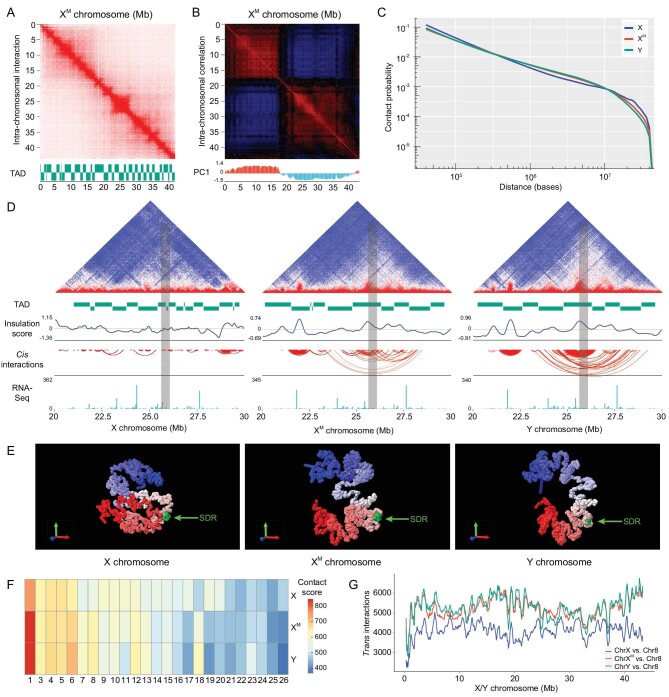
Plastic chromatin 3D structure with dynamic gene expression of sex chromosomes. (A) Hi-C interaction heat maps (50-kb resolution) of the X^M^ chromosome. Identified TADs are shown at the bottom. (B) Correlation heat maps (100-kb resolution) of the X^M^ chromosome. Under the heat map, we show the PC1 score of the Hi-C interaction matrix. (C) The dependence of the contact probability on the genomic distance averaged over the X, X^M^ and Y chromosomes. (D) Epigenetic profiles covering 20- to 30-Mb regions of the X, X^M^ and Y chromosomes were captured. The top panel shows the Hi-C heat map at a 40-kb resolution. The first panel in the lower part shows the identified TADs. The second panel shows the insulation score. The third panel shows the significant *cis* interactions identified. The last panel shows the RNA-Seq coverage with a 50-kb sliding window. The SDR is highlighted in gray. (E) 3D conformations of the X, X^M^ and Y chromosomes. The SDR is highlighted in green. Blue to red represent the 5' end to 3' ends of the chromosome sequence, respectively. (F) Comparison of the positions of X, X^M^ and Y relative to autosomes. Higher contact scores indicate closer proximity. (G) An example showing the spatial proximity between the X, X^M^ and Y chromosomes with Chromosome 8. *Trans* interactions between sex chromosomes and Chromosome 8 were counted using a 500-kb sliding window with a step of 100 kb.

Parallel Hi-C experiments were performed in the gonad cells of sex-reversed XX-neomale yellow catfish at the same time and using the same protocol as in XX-female and YY-supermale yellow catfish. As a result, ∼550 million read pairs and ∼254 million valid read pairs were obtained after filtering. Interestingly, we found that the Hi-C contact map of the neomale X chromosome (X^M^ chromosome) exhibited very high similarity to the Y chromosome, with a Pearson correlation of 0.99 (Fig. 5A and Supplementary Fig. 20). Forty-three TADs were identified on the X^M^ chromosome, 38 (88.4%) of which shared boundaries with the Y chromosome (Fig. 5A). Similar chromosomal compartmentalization was observed between the Y chromosome and the X^M^ chromosome; i.e. the X^M^ chromosome is also divided into one large A compartment and one large B compartment (Fig. 5B). We then examined how contact probability depended on genomic distance on the X, X^M^ and Y chromosomes. Compared with the X chromosome, both the X^M^ and Y chromosomes exhibited an increased frequency of mid-range (300 kb–10 Mb) interactions and decreased frequencies of short- (<300 kb) and long-range (>10 Mb) interactions, which also indicated that the X chromosome structure was denser than those of the X^M^ and Y chromosomes (Fig. 5C).

To verify whether similar chromatin structures correspond to similar gene expression, we compared the gonad transcriptome data of the X, X^M^ and Y chromosomes. Both the relative gene expressions on the X^M^ and Y chromosomes were higher than that of the X chromosome (*P*-value = 5.738 × 10^−11^ and 4.813 × 10^−13^, Wilcoxon test), consistently with their looser chromatin structures (Supplementary Fig. 21). Only 31 differentially expressed genes (DEGs) were identified between the X^M^ and Y chromosomes, while 498 DEGs and 540 DEGs were identified in the Y chromosome vs X chromosome and X^M^ chromosome vs X chromosome comparisons, respectively (Supplementary Table 5). In addition, the great majority of the sex-biased DEGs identified between the Y chromosome and the X chromosome overlapped with the sex-biased DEGs between the X^M^ chromosome and Y chromosome (male bias, 269 out of 331; female bias, 196 out of 242). This was consistent with the heat-map and hierarchical-clustering analyses performed on sex-biased genes of the X, X^M^ and Y chromosomes, which showed a group of phenotypic males (XX males, YY supermales) clearly separated from the phenotypic female-sex-chromosome (XX females) group (Supplementary Fig. 22).

We then investigated the local chromatin organization of the X, X^M^ and Y chromosomes. The SDR is located in a large shared TAD (24.80–26.28 Mb) on the X^M^ and Y chromosomes and across three small TADs on the X chromosome (Fig. 5D). Furthermore, by inspecting significant *cis* interactions, we observed more long-range interactions on the X^M^ and Y chromosomes than on the X chromosome, where the SDR-related TAD contains many significant *cis* interactions and is located in a large loop-like domain (23–29 Mb) (Fig. 5D). Furthermore, the reconstructed 3D structures from different view angles clearly revealed the spatial differences between the X, X^M^ and Y chromosomes (Fig. 5E and Supplementary Fig. 23). The 3D structure of the X^M^ chromosome is more similar to that of the Y chromosome (RMSE = 1.02 × 10^−4^, spearman correlation coefficient = 0.864) compared to the X chromosome (RMSE = 1.89 × 10^−4^, spearman correlation coefficient = 0.574). The X chromosome displayed a more compact structure relative to the looser structures of the X^M^ and Y chromosomes. In addition, the SDR could be identified in a large loop structure shared by X^M^ and Y chromosomes, while it was not found on the X chromosome (Fig. 5E and Supplementary Fig. 24).

To test whether sex reversal shifted the spatial arrangement of chromosomes in the nucleus, we used contact scores to assess the spatial proximity between chromosomes in XX females, XX males and YY supermales. We noted that the smaller-sized chromosomes presented more frequent *trans* contacts with each other, suggesting that they may be spatially close to each other (Supplementary Fig. 25). As expected, females and males showed overall different spatial organizations. For example, after sex reversal, the contact score between Chromosomes 25 and 26 decreased (XX female: 13 731.38; XX neomale: 12 195.27), while the contact score between Chromosomes 23 and 24 increased (XX female: 12 404.38; XX neomale: 12 759.22). Additionally, XX neomales and YY supermales showed nearly identical spatial arrangements with a Pearson correlation coefficient of 0.994 (Supplementary Fig. 26). Then, we examined the spatial proximity between the three kinds of sex chromosomes and autosomes. The spatial arrangement of the X^M^ chromosome was highly consistent with that of the Y chromosome, with a Pearson correlation coefficient of 0.978 (*P*-value < 2.2 × 10^−16^). Interestingly, the X^M^ chromosome showed a more similar spatial arrangement to the X chromosome (Pearson correlation coefficient = 0.859, *P*-value = 3.788 × 10^−8^) than to the Y chromosome (Pearson correlation coefficient = 0.778, *P*-value = 4.681 × 10^−6^) (Fig. 5F and Supplementary Fig. 27). Furthermore, the patterns of spatial proximity between the three kinds of sex chromosomes and Chromosome 8 clearly revealed stronger interactions of the X^M^ and Y chromosomes with the autosome (Fig. 5G).

## DISCUSSION

In this study, we successfully assembled highly homomorphic X and Y chromosomes by sequencing the genomes of YY-supermale and XX-female yellow catfish and discovered a new class of XX/XY sex chromosomes that originated by autosome–autosome fusion. Although the Y and X chromosomes had high sequence identity and showed no chromosome inversions or translocations, specific accumulation and distribution patterns of repetitive DNA sequences were observed on the Y chromosome. We further inferred that sex reversal could cause chromatin remodeling of X chromosomes of XX neomales and make it similar to that of the Y chromosome in YY supermales. These data might help reveal the origin and evolution of sex chromosomes and sexual plasticity in fish and amphibians.

X and Y chromosomes are highly differentiated in mammals but apparently homomorphic in most fish species, such as yellow catfish, in which the sequence divergence is only <1% and gene compositions are the same between X and Y chromosomes. Rearrangements were considered as an effective way to reduce the recombination rate [49–51]. However, there are no classical structure features to suppress the recombination between X and Y chromosomes in yellow catfish, only minor accumulation of indels and some repetitive elements. Research on sex chromosomes without rearrangements suggested that the progressive development of recombination arrest is an alternative mechanism [52,53]. Repetitive elements, especially transposons, have been shown to play a central role in this process by causing insertion and duplication [50,54–56]. A small SDR (196 kb) was identified in turquoise killifish (*Nothobranchius fuzeri*), in which neither inversions nor candidate sexually antagonistic genes were found, but a 241-bp deletion may be the main cause of recombination suppression [57]. Several recent studies have shown that the appearance of inversions and translocations is facilitated by the reduced recombination rates, which means the accumulation of rearrangements may be a consequence instead of a cause [11,48]. Therefore, the indels and transposons enriched in the SDR might be the main driver of initial restricted recombination suppression between young X and Y chromosomes in fish species, such as yellow catfish. Almost all sex-specific SNPs were located in the SDR of yellow catfish (Fig. 1) and 76.5% of these male-specific SNPs were located in *pfpdz1*, which is essential for male-sex differentiation and maintenance [58]. There are some Y-specific DNA transposons and retrotransposons in *pfpdz1* gene (Supplementary Fig. 12), while transposon-induced epigenetic modification has been shown to regulate sex determination in the fighting fish [59].

**Figure 6.**
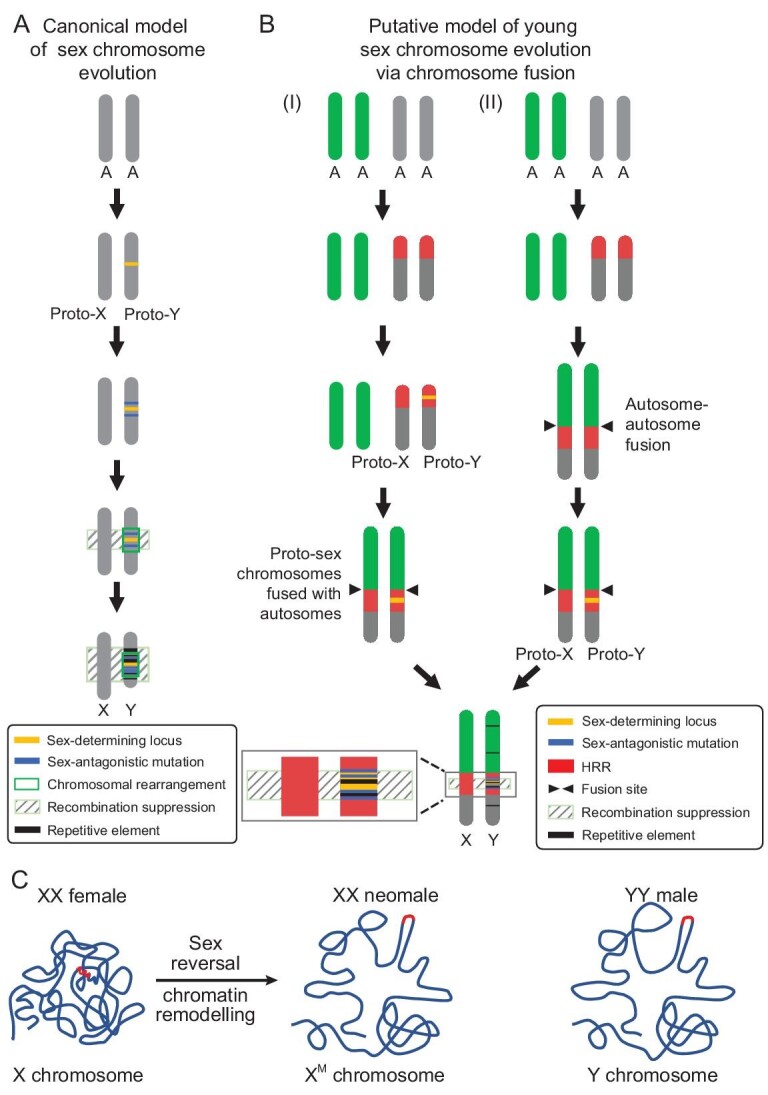
Putative steps involved in the evolution of young sex chromosomes and their chromatin remodeling in yellow catfish with sexual plasticity. (A) The canonical model of X- and Y-chromosomes evolution. (B) The origin and evolution of homomorphic young sex chromosomes via autosome–autosome fusion in yellow catfish. Two hypothesized models were proposed. (C) The chromatin organization of sex chromosomes in yellow catfish. The SDRs are highlighted in red.

Chromosome fusion could advance the speciation process by establishing barriers to gene flow [60–62] and a lower chromosome number of yellow catfish compared to redtail catfish, channel catfish and southern catfish does provide evidence for chromosome fusions. Fusion between autosomes and existing Y chromosomes could create an X1 × 2Y system, with the unfused homologous segregating as a neo-X chromosome [40]. However, to date, there are no reports of females and males with different chromosome numbers in Siluriformes. Sex-chromosome turnovers in fishes are prevalent, which are usually caused by the creation of a new sex-determining gene on an autosome [63,64] or transposition of a sex-determining locus to an autosome [65,66]. Different sex chromosomes in southern catfish, channel catfish, redtail catfish and yellow catfish suggest that sex-chromosome turnovers might occurred frequently in Siluriformes (Fig. 3A). In willows, repeated turnovers lead to restricted degeneration, keeping the sex chromosomes in a permanent state of youth [67], which might be a plausible explanation for the prevalent homomorphic sex chromosomes in Siluriformes. A HRR was observed downstream of the fusion site in yellow catfish and the SDR is located in the middle of this region with already suppressed recombination (Fig. 3). Relative to other chromosomes, there are significantly more rearrangement events in the HRR region, which were considered as a source of variation in gene diversity [68]. It has been found that the chromosomal rearrangements of the genome and the emergence of sex chromosomes are coupled in cichlids [21], which suggests that the emergence of chromosomal rearrangements could be a potential driver of neo-sex-chromosome formation. Evolutionary strata have been observed on the sex chromosomes of humans [3], chickens [69], snakes [70] and some fish including three-spined stickleback [39] and spotted knifejaw [40]. In contrast, the sex chromosomes of yellow catfish did not show identifiable evolutionary strata, which indicates that the sex chromosomes of yellow catfish still remain at an early stage of sex-chromosome evolution. Subsequently, on the basis of the canonical model (Fig. 6A), we proposed two hypotheses for the evolution of sex chromosomes via chromosome fusion in yellow catfish (Fig. 6B). In the initial stages of both hypotheses, the emergence of HRRs on a pair of autosomes that resulted from several large genome-arrangement events facilitates speciation. Next, in the first hypothesis, this autosome pair might be recruited as proto-sex chromosomes after the acquisition of a sex-determining locus through diversification of a pre-existing locus in the HRR. Immediately afterwards, the transitional sex chromosomes might recruit another autosome pair to result in neo-sex chromosomes via chromosome fusion. Alternatively, the second hypothesis suggests that the emergence of proto-sex chromosomes might occur after autosome–autosome fusion. The driving forces behind these fusions are worthy of further studies. The inconsistent aspect between our hypotheses with the classical model is that the SDR-restricted recombination suppression is initiated by accumulation of repetitive elements and sex-antagonistic mutations, rather than by inversions or translocations.

Recently, epigenetic modifications, including genomic DNA methylation and histone modification, have been confirmed to control sex determination by regulating the expression of sex-determining genes in fish species and reptiles [24–26]. Epigenetic modifications control gene transcriptional regulation by orchestrating the 3D chromatin organization, which physically connects distant *cis*-regulatory elements with gene promoters through chromatin looping and compartmentalization [28,71]. However, the 3D chromatin organization of the X and Y chromosomes is unknown due to the lack of high-quality reference sequences. Although the X and Y chromosomes of yellow catfish show high sequence identity, the Y chromosome of YY supermales exhibits higher levels of chromosome organization than the X chromosome of XX females, including a larger size of TADs and a highly organized A/B compartment. The compartmental changes correspond to gene-expression levels on the X and Y chromosomes (Figs 4 and 5), which may be due to the divergent distribution of repetitive elements on the X and Y chromosomes, since repeat elements have been shown to organize the 3D genome structure and regulate gene transcription [72,73]. Chromatin loops can bring the regulatory elements and their distant targets together to a closer physical proximity [74,75]. A loop-like structure containing the SDR was observed in Y chromosome but not in X chromosome (Fig. 5C and Supplementary Fig. 24), which might regulate the male-biased expression of candidate sex-determining genes in yellow catfish.

Sex determination in fish species and amphibians is plastic and modulated by both genetic factors (GSD) and some environmental factors (ESD), such as temperature, exogenous hormones and chemicals [22]. Moreover, environmental factors could induce epigenetic modifications and chromatin remodeling, thereby regulating patterns of gene expression [27,28,76–78]. Sex reversal triggers the rapid transition from GSD to TSD in Australian bearded dragon (*Pogona vitticeps*) and yellow catfish [29,30]. The interaction between GSD and ESD is an important driver of sex-chromosome evolution [79]. XX-neomale yellow catfish were artificially produced by treatment with letrozole, a chemical of aromatase inhibitor [30]. The 3D chromatin organization of the sex chromosomes as well as the nucleus spatial organization of XX neomales were remodeled after sex reversal and similar to those in YY supermales. Meanwhile, gene expression and a male-specific loop containing the SDR in XX neomales are extremely similar to YY supermales. Open chromatin conformation plays a key role in the precise regulation of gene expression [28,71]. Compared with the highly condensed sex chromosomes of XX females, the open chromatin structure of X^M^ and Y chromosomes allows the SDR and sex-determining genes to be more easily accessed and activated (Figs 5E and 6C). Spatial organizations in the nucleus play an important role in the regulation of nuclear function [80–82]. Our results showed that sex chromosomes may regulate the expression of sex-related genes in different chromosomes through spatially closing to them, especially their promoters (Figs 4F and G, and 5F).

Overall, in the current study, we assembled the X and Y chromosomes of yellow catfish and provided genomic evidence that the sex-chromosome pair of the fish species is still at the initial stage of evolution. The sex chromosomes of yellow catfish originated from the fusion of two autosomes. The slight accumulation of indels and some types of repetitive elements might play an important role in the initial establishment of recombination suppression and sex-chromosome differentiation. The 3D chromatin conformation of the Y chromosome is associated with sex-specific gene expression and sex determination, while changes in the 3D chromatin structure mediated by genetic and environmental factors are suggested to drive sexual plasticity in lower vertebrates.

## MATERIALS AND METHODS

### Fish sources

All yellow catfish used in this study were reared at the breeding centre of Huazhong Agricultural University in Wuhan City, Hubei Province. All experiments involving yellow catfish were approved and performed in compliance with the requirements of the IACUC of Huazhong Agricultural University (HZAUFI-2017-003).

### Metaphase preparation and FISH

Chromosome preparation and FISH analysis were performed as previously described [83,84]. BAC clones that contain sex-linked markers have been identified and used for DNA isolation [36]. BAC DNA labeled with DIG-Nick Translation Mix (Roche) was used as the FISH probe. Cells and metaphase chromosomes on slides were denatured in 70% deionized formamide/2 × saline-sodium citrate buffer (SSC) for 4 min at 72°C. For hybridization, a 50-μl hybridization mixture containing 100 ng of labeled probe, 50% formamide, 20% dextran sulfate, 0.5 μg/μl sheared salmon sperm DNA (sssDNA), 0.1% sodium dodecyl sulfate and 2 × SSC was denatured at 95°C for 5 min and then added to target slides and covered with a 24 × 50 mm^2^ coverslip to spread the hybridization solution. The hybridization reaction was performed in a wet box at 37°C for 24–48 hours. After a series of post-hybridization washes, the slides were co-incubated with a fluorescein isothiocyanate (FITC)-conjugated anti-digoxigenin antibody (Roche) and diamidino-phenyl-indole (DAPI) for 1 hour, and the fluorescent signals were detected and captured under a ×63 oil lens.

### Experimental procedures and data analysis of genome sequencing, Hi-C and RNA-seq

Detailed descriptions of experimental procedures and data analysis are available in the Supplementary data.

### Quantitative real-time PCR (qRT-PCR)

After determining the genotypic sex of XX female, XX neomale and YY supermale by sex-linked markers [36], an XX-female population and an XY-male population were produced by crossing XX males and YY supermales with XX females. Gonads of 30 XX females and 30 XY males were collected at 10, 15, 20, 25 and 30 days post-hatching (dph), respectively. RNA was extracted using TRIzol reagent (Invitrogen, USA) and transcribed into cDNA using a PrimeScript RT reagent kit (Takara, Japan). qRT-PCR was performed using 2X Universal SYBR Green Fast qPCR mix (ABclonal, China) on a Bio-Rad CFX96 thermal cycler (Bio-Rad, USA) as previously described [85]. The data were analysed using the 2^−ΔΔCt^ method and the expression levels of the target gene were normalized using the housekeeping control gene *β-actin*. The gene-specific primer sequences are listed in Supplementary Table S6.

### Statistical analysis and reproducibility

Statistical analyses were carried out using R and all figures were plotted in R [86].

## DATA AVAILABILITY

The XX and YY yellow catfish assemblies from this study have been submitted to the NCBI Nucleotide database (https://www.ncbi.nlm.nih.gov/nuccore/) under accession numbers JAJLOY000000000 and JALFZK01, respectively. The raw sequencing reads generated in this study have been submitted to the NCBI BioProject database (https://www.ncbi.nlm.nih.gov/bioproject/) under accession number PRJNA748903.

## Supplementary Material

nwac239_Supplemental_Files
