# Supplementary material for: Origin and chromatin remodeling of young X/Y sex chromosomes in catfish with sexual plasticity
Source: Natl Sci Rev. 2022 Oct 28;10(2):nwac239. doi: 10.1093/nsr/nwac239 (PMC9945428; doi:10.1093/nsr/nwac239)
Supplement: nwac239_Supplemental_Files [file nwac239_supplemental_files.zip › Supplementary_File.docx]

**SUPPLEMENTAL INFORMATION FOR**

**Origin and chromatin remodelling of young X/Y sex chromosomes in catfish with sexual plasticity**

**Authors:**

Gaorui Gong^1^, Yang Xiong^1^, Shijun Xiao^3^, Xi-Yin Li^2^, Peipei Huang^1,4^, Qian Liao^1^, Qingqing Han^1^, Qiaohong Lin^1,2^, Cheng Dan^2^, Li Zhou^2^, Fan Ren^1^, Qi Zhou^5^, Jian-Fang Gui^1,2*^, Jie Mei^1,*^

**Affiliations:**

^1^Hubei Hongshan Laboratory, College of Fisheries, Huazhong Agricultural University, Wuhan, 430070, China.

^2^State Key Laboratory of Freshwater Ecology and Biotechnology, Hubei Hongshan Laboratory, Institute of Hydrobiology, Chinese Academy of Sciences, University of the Chinese Academy of Sciences, Wuhan, 430072, China.

^3^Jiaxing Key Laboratory for New Germplasm Breeding of Economic Mycology, Jiaxing, 314000, China

^4^School of Animal Science and Nutritional Engineering, Wuhan Polytechnic University, Wuhan, 430023, China.

^5^MOE Laboratory of Biosystems Homeostasis & Protection, Life Sciences Institute, Zhejiang University, Hangzhou, 310058, China.

**Methods**

**Sample collection and whole genome sequencing**

A YY genotype male yellow catfish was used to prepare DNA for genome sequencing. DNA for sequencing was isolated from muscle using the phenol/chloroform extraction method as described in a previous study (1). The quality of the DNA was checked by agarose gel electrophoresis. Three 20-kb SMRTbell libraries were constructed as previously described (1) and sequenced on the PacBio RS II platform (Pacific Biosciences, USA). For short read sequencing, the genomic DNA was fragmented into a fragment size of ~350 bp and the paired-end genomic library was prepared following the manufacturer’s protocols (Illumina, USA). Then, the genomic library was sequenced on an Illumina HiSeq X-Ten sequencing platform with paired-end 150 bp read layout.

To prepare the Hi-C library for genome scaffolding, blood samples from the same yellow catfish used for genomic DNA sequencing were extracted. The Hi-C experiment consisted of cell crosslinking, cell lysis, chromatin digestion, biotin labelling, proximal chromatin DNA ligation and DNA purification, which were performed following previously reported procedures (1). Finally, the Hi-C libraries were quantified and sequenced on the Illumina HiSeq X-Ten platform using a PE-150 module.

**Genome assembly and annotation**

First, Flye version 2.6 (2) was employed to assemble the YY supermale PacBio raw reads with the parameters ‘--pacbio-raw -g 720m --iterations 2’. Second, wtdbg2 version 2.5 (3) was used separately for genome assembly with the default parameters. We mapped the PacBio sequencing reads to the draft contigs generated by Flye and wtdbg2 using pbalign and polished the resulting contigs using Quiver (4) with the Arrow algorithm. As Illumina data are known to show a lower overall error rate than PacBio data, two rounds of consensus correction were performed using Illumina reads mapped with BWA version 0.7.17 (5) and NextPolish version 1.1.0 (6). The Flye assembly was scaffolded with Hi-C data using the 3D-DNA pipeline (7). The Hi-C reads were aligned to the Flye polished contigs using the Juicer pipeline (8). The 3D-DNA pipeline was run with the default parameters. The results were manually reviewed using Juicebox assembly tools (9) until the overall heatmap conformed to the characteristics of chromosome interactions. To take advantage of the sequence complementation of the two assemblies from Flye and wtdbg2, the wtdbg2 assembly was transformed into long overlapping sequences with a maximum of 15 kb (500 bp overlap). Then, the result was used as the input for TGS-GapCloser (10) with the default parameters to fill the gaps in the Hi-C scaffolds obtained from 3D-DNA. Protein-coding genes and repeat annotation were performed using the same methods as in a previous study (1). To annotate putative centromeres, TRFinder version 4.09 (11) was employed to search tandem repeats across the genome with the following parameters: 2 5 7 80 10 50 2000. Then, repeat unit redundancy was removed using pyTanFinder (12). Finally, we overlapped these findings with regions that tended to show low Hi-C contact with identified putative centromeric regions.

The Illumina and PacBio sequencing data of XX female yellow catfish were obtained from a previous study (NCBI Bioproject: PRJNA489116). The XX genome was assembled and annotated using the same method as above. The assembly quality was assessed by comparison with BUSCO version 3.1.0 (13) with the “actinopterygii_odb9” dataset.

**Pooled genome sequencing (Pool-Seq), whole genome resequencing and sex determining region identification**

Genomic DNA was extracted from the fin clips of one family of XX female (n=20) and one family of YY supermale yellow catfish (n=20) and used for the Pool-Seq analysis. The extracted genomic DNA was pooled at an equimolar ratio according to sex, and Pool-seq libraries were generated using the TruSeq Nano DNA Sample Prep Kit (Illumina, ref. FC-121-4001) following manufacturer’s instructions. Then the Pool-Seq libraries were sequenced on the Illumina HiSeq X-Ten platform in PE-150 mode. For whole genome resequencing, genomic DNA of another two family of XX female (n=19) and YY male (n=19) individuals were extracted, respectively. The DNA library of each individual was prepared with the MGIEasy FS DNA Prep Set (MGI, China) and sequenced on the MGI DNBSEQ-T7 platform in PE-150 mode.

The sequencing reads of each library were filtered using fastp version 0.19.7 (14) with the default parameters. The treated cleaned reads from the male and female pools were remapped to the XX genome sequence using BWA-MEM version 0.7.17 with the default parameters. The generated BAM files were further processed via the GATK pipeline (15) to call variants. Popoolation2 (16) was used to calculate F_ST_ values based on allele frequency differences between the male and female pools. To obtain sex-specific variants, a custom Python script was used to identify homozygous mutant variants in the male pool and 19 male individuals that were fixed in the female pool and 19 female individuals at the same position. We used a 20-kb sliding window with a step of 5 kb to count these sex-specific variants.

To identify the sex chromosomes, a sex chromosome BAC sequence was obtained from a previous study (17) and was aligned against the XX and YY assemblies using BLAT (18).

**Comparative genomic analysis**

The YY yellow catfish assembly was aligned with the XX yellow catfish assembly using LAST version 1047 (19). Locally collinear blocks between chromosomes X and Y were identified by using Mauve version 2.3.1 (20). A collinearity analysis between redtail catfish (*Mystus wyckioides*), southern catfish (*Silurus meridionalis*), channel catfish (*Ictalurus punctatus*) and yellow catfish was performed using the MCscan toolkit implemented in Python (https://github.com/tanghaibao/jcvi) (21). Genome sequence alignments between the X/Y chromosomes of yellow catfish and corresponding chromosomes of southern catfish, channel catfish and yellow catfish were performed using Minimap2 (22) and visualized using D-Genies (23). Genome sequence alignments between the X/Y chromosome of yellow catfish and the corresponding chromosomes of southern catfish, channel catfish and yellow catfish were performed using Minimap2 (22) and visualized using D-Genies (23). Ka and Ks values were calculated for each pairwise alignment using the CodeML module of PAML (Phylogenetic Analysis by Maximum Likelihood) (24).

**Recombination rate estimation**

ReLERNN version 1.0.0 (25) was employed to estimate recombination rates using the Pool-seq data with the default parameters of Pool-seq mode. Briefly, ReLERNN takes advantage of a recurrent neural network to infer the genome-wide landscape of recombination from as few as four individually sequenced chromosomes or from allele frequencies inferred by pooled sequencing.

**Hi-C sequencing and comparative analysis**

Gonads of an XX male, a YY supermale and an XX neomale yellow catfish were harvested for Hi-C library preparation. For each sample, a Hi-C library was prepared using an *in situ* protocol (26). All libraries were sequenced on the Illumina HiSeq X-Ten platform in PE-150 mode.

Hi-C reads mapping, filtering, correction, binning and normalization were performed by using HiC-Pro version 2.11.3 (27) with the default parameters. Briefly, the sequencing reads from YY supermale were mapped to the YY genome, and the sequencing reads from XX female and XX neomale were mapped to the XX genome. Then, uniquely mapped reads were retained and assigned to a restriction enzyme fragment, and invalid ligation products were discarded. The interaction contacts were binned to generate the genome-wide interaction matrix at a 10-kb, 20-kb, 40-kb, 100-kb and 500-kb resolution. The interaction matrix was normalized via the iterative correction and eigenvector decomposition (ICE) method (28). Using the obtained valid reads, the contact score between chromosome *i* and chromosome *j* (*C_ij_*) is defined as follows:

$$C_{ij}= \frac{N_{ij}}{\left( L_{i}+L_{j} \right)\times T}$$

Here, *N_ij_* is the number of *trans* interactions between chromosome *i* and chromosome *j*; *L_i_* and *L_j_* denote the length of chromosome *i* and chromosome *j*, respectively; and *T* denotes the total number of *trans* interactions in each sample. The significant *cis* interactions and *trans* interactions were calculated using FitHiC version 2.0.7 (29) at a 20-kb resolution with an adjusted *p* value < 0.05. The regions showing significant *trans* interactions with sex chromosomes were annotated using ChIPseeker (30). KOBAS (31) was employed to perform KEGG enrichment, and Metascape (32) was employed to perform GO enrichment. A/B compartments were identified using the runHiCpca.pl script of the HOMER package (http://biowhat.ucsd.edu/homer) with the parameters “-res 100000”. TADs were identified using the insulation score described in a previous study with the default parameters (33). The sex chromosome 3D forms of three samples were reconstructed using the LorDG algorithm (34) and visualized using GenomeFlow version 2.0 (35).

**mRNA sequencing and gene expression analysis**

The testes of three XX neomales and three YY supermales and the ovaries of three XX male yellow catfish were collected for RNA sequencing. Total RNA was extracted using the RNeasy Kit (Qiagen, USA) according to the manufacturer’s instructions. mRNA was purified from total RNA as described previously using the NEBNext® Ultra II Directional RNA Library Prep Kit for Illumina (NEB, E7760) according to the manufacturer’s instructions. Finally, all libraries were sequenced on the Illumina HiSeq 2000 platform with a paired-end 150-bp read length.

Raw paired-end reads were filtered using fastp version 0.19.7 under the default parameters. Filtered reads were mapped to the yellow catfish genome using STAR version 2.7.2b (36) with the following command line options “—twopassMode Basic –outFilterMultimapNmax 10 –quantMode TranscriptomeSAM GeneCounts”. Gene-level quantification was performed using RSEM version 1.3.1 with the default parameters (37). The R package DESeq2 (38) was employed to evaluate differential gene expression. Genes with an absolute log_2_(fold-change) value ≥ 2 and adjusted *p* value < 0.05 were defined as DEGs.

**Supplementary figures and figure legends**

**
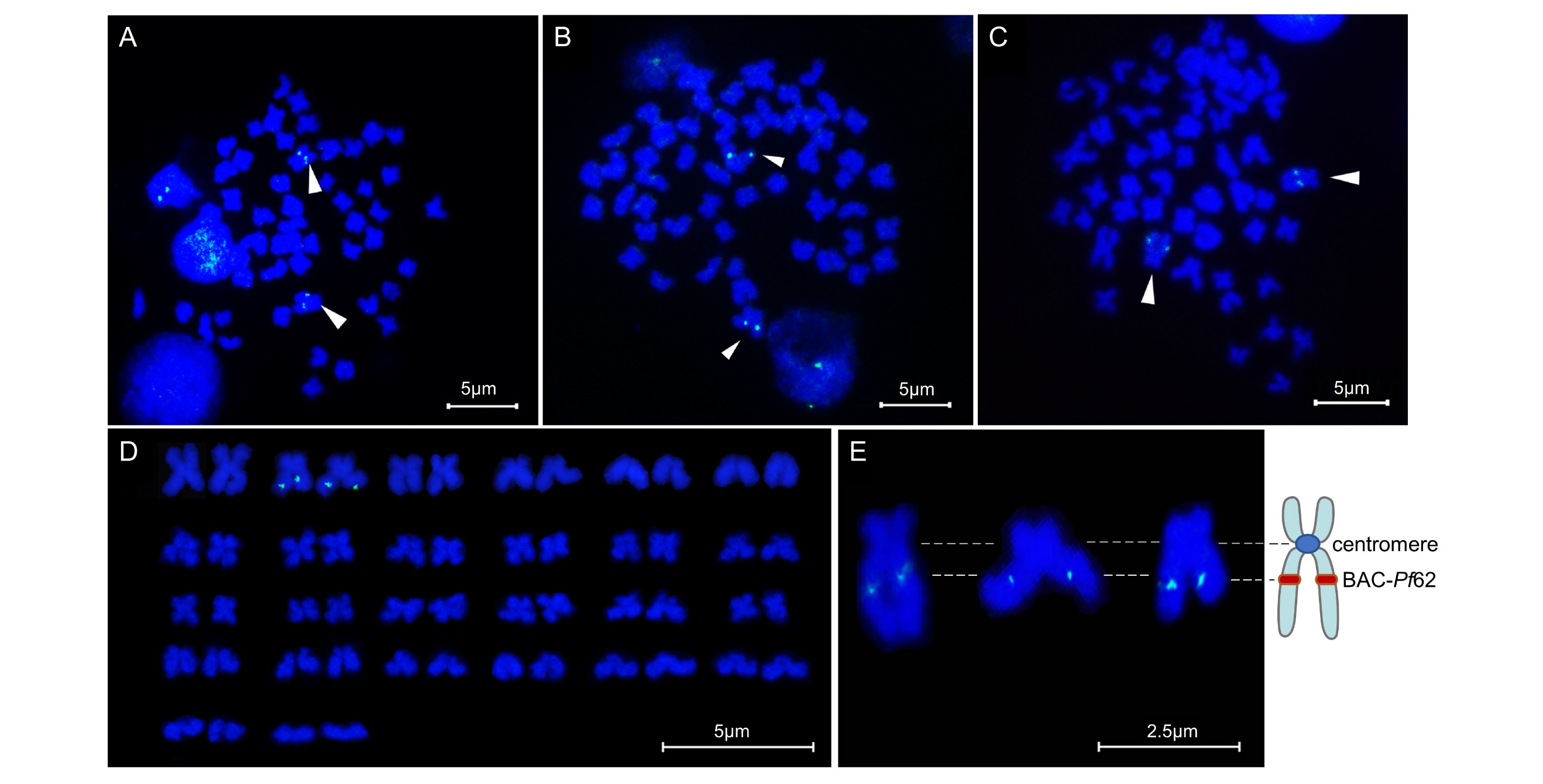
**

**Supplementary Fig. 1. Identification of sex chromosomes by BAC-FISH.**

(A-C) Hybridization signal detection in metaphase cells of XY- (A), XX- (B) and YY- (C) genotype yellow catfish. Signals are indicated with white arrowheads. (D) Karyotyping analysis of XY male yellow catfish by DAPI staining. The sex chromosome pair shows the second largest size of all chromosome pairs. (E) Morphological and structural characterization of the submetacentric sex chromosome at a magnified level, and the structural schema is shown on the right.

**
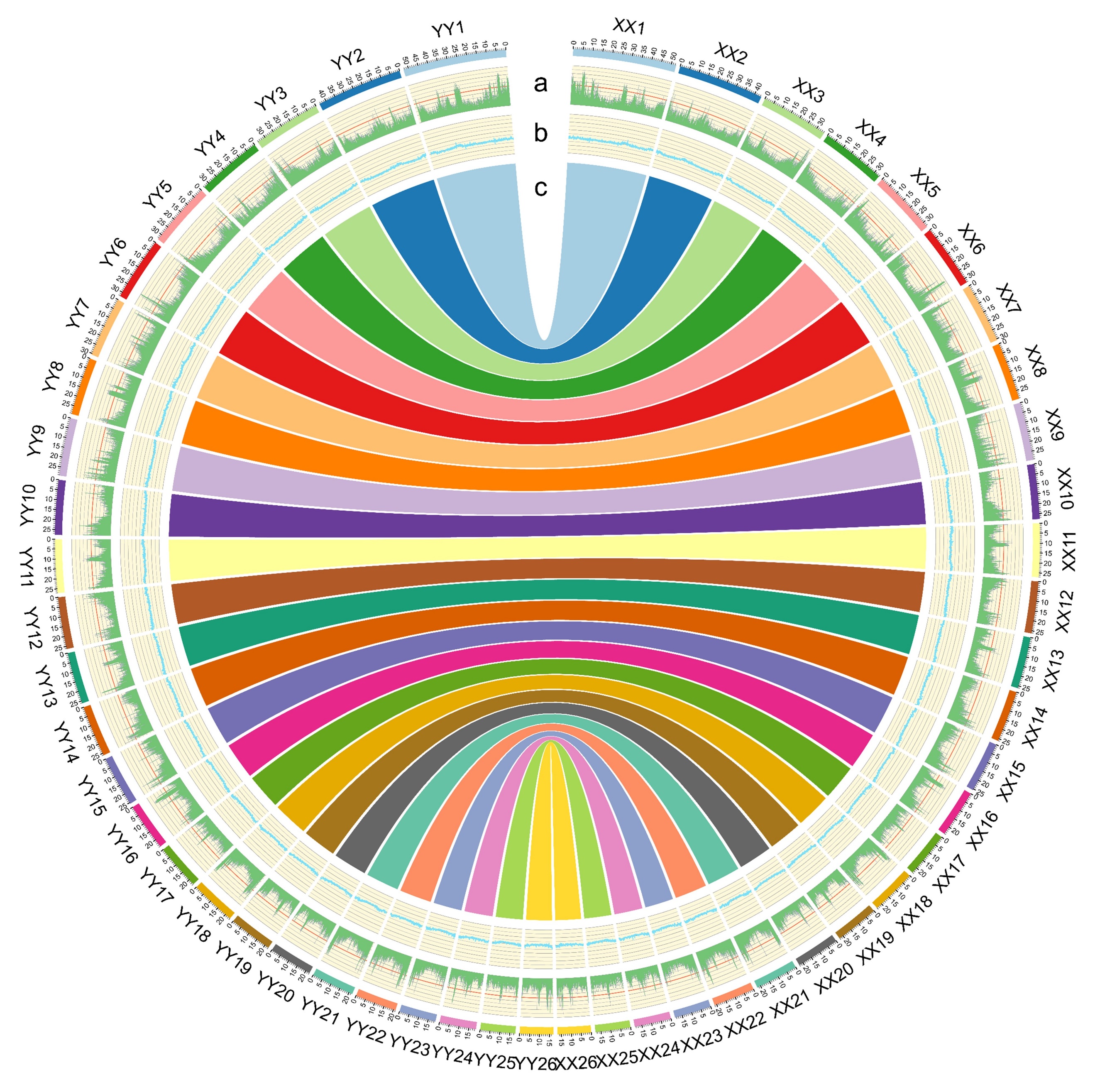
**

**Supplementary Fig. 2. Synteny and whole-genome alignment between XX and YY yellow catfish.** The arcs of concentric circles (c) represent each chromosome of the XX female yellow catfish genome and YY male yellow catfish genome. The repeat content (a) and GC content (b) of each chromosome are shown from the outside to the inside.


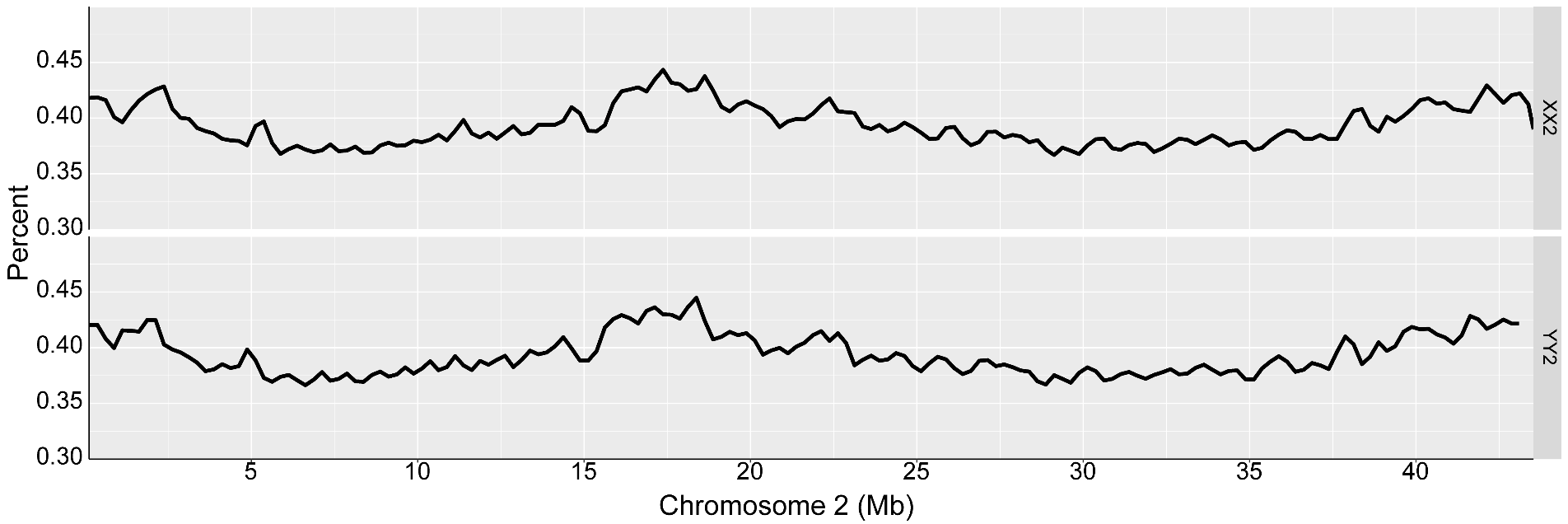


**Supplementary Fig. 3. Distribution of GC content on the X and Y chromosomes.**


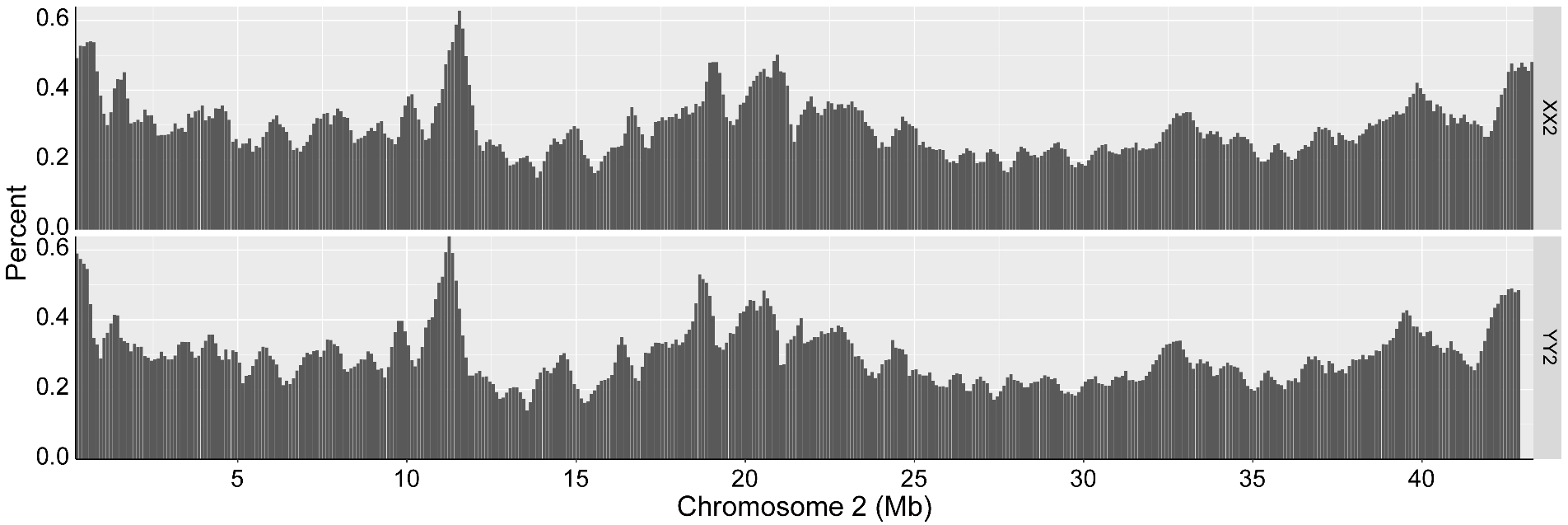


**Supplementary Fig. 4. Distribution of repeat content on the X and Y chromosomes.**


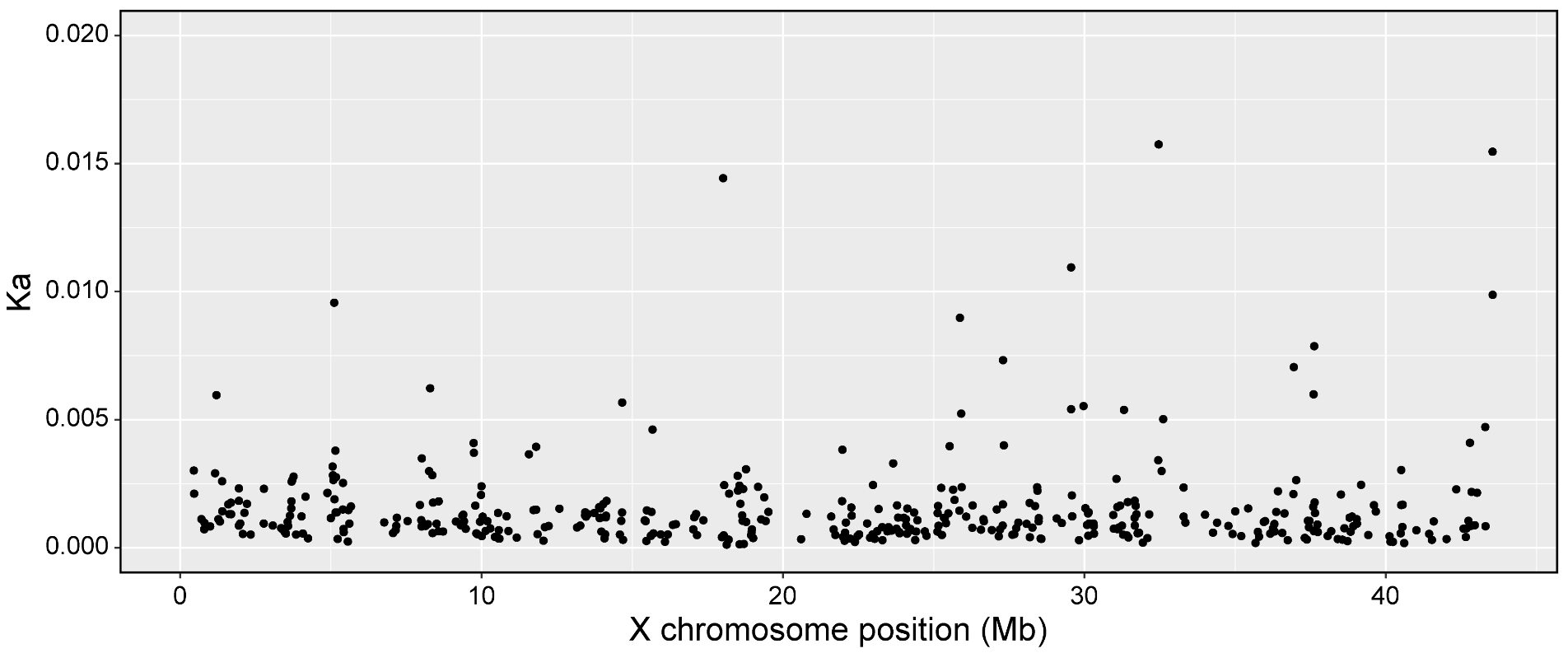


**Supplementary Fig. 5. Non****synonymous divergence (Ka) between the X and Y chromosomes was estimated for every annotated gene.** Genes are ordered by their position on the X chromosome.


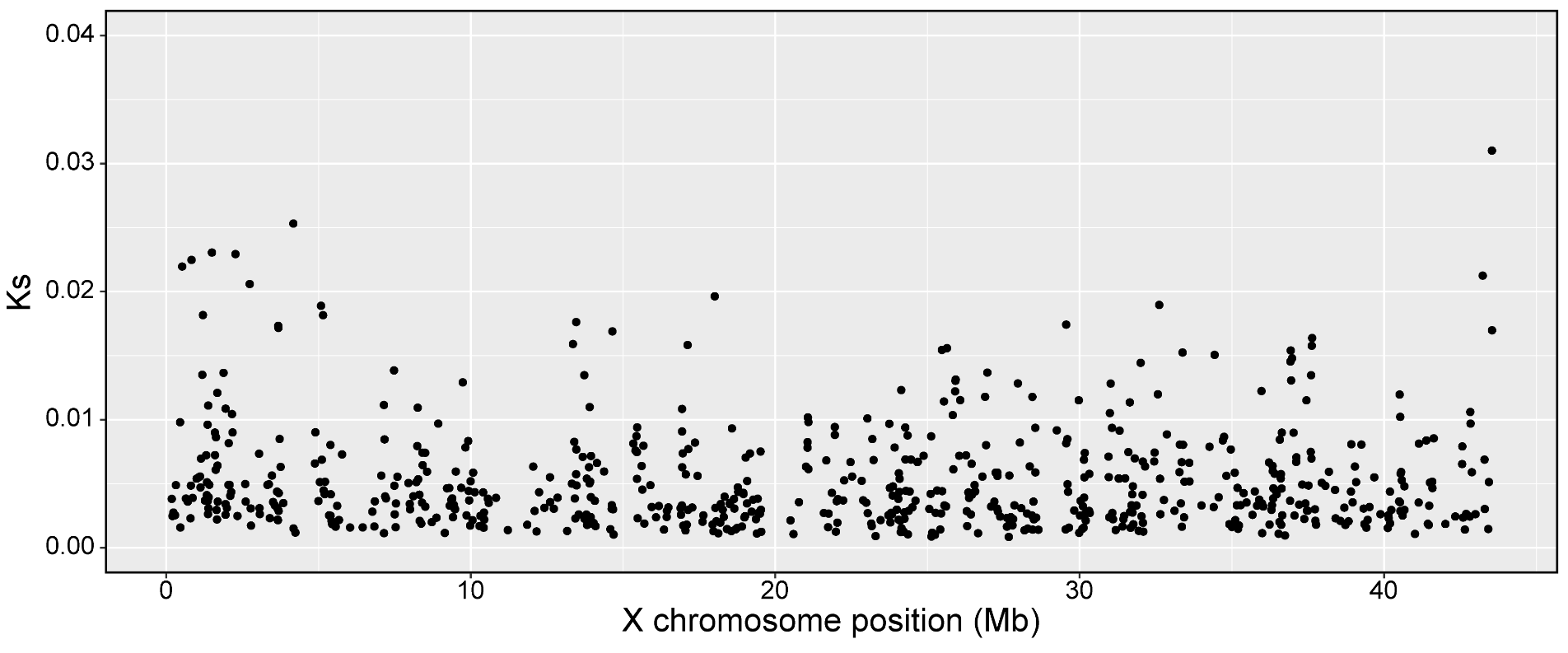


**Supplementary Fig. 6. Synonymous divergence (Ks) between the X and Y chromosomes was estimated for every annotated gene.** Genes are ordered by their position on the X chromosome.


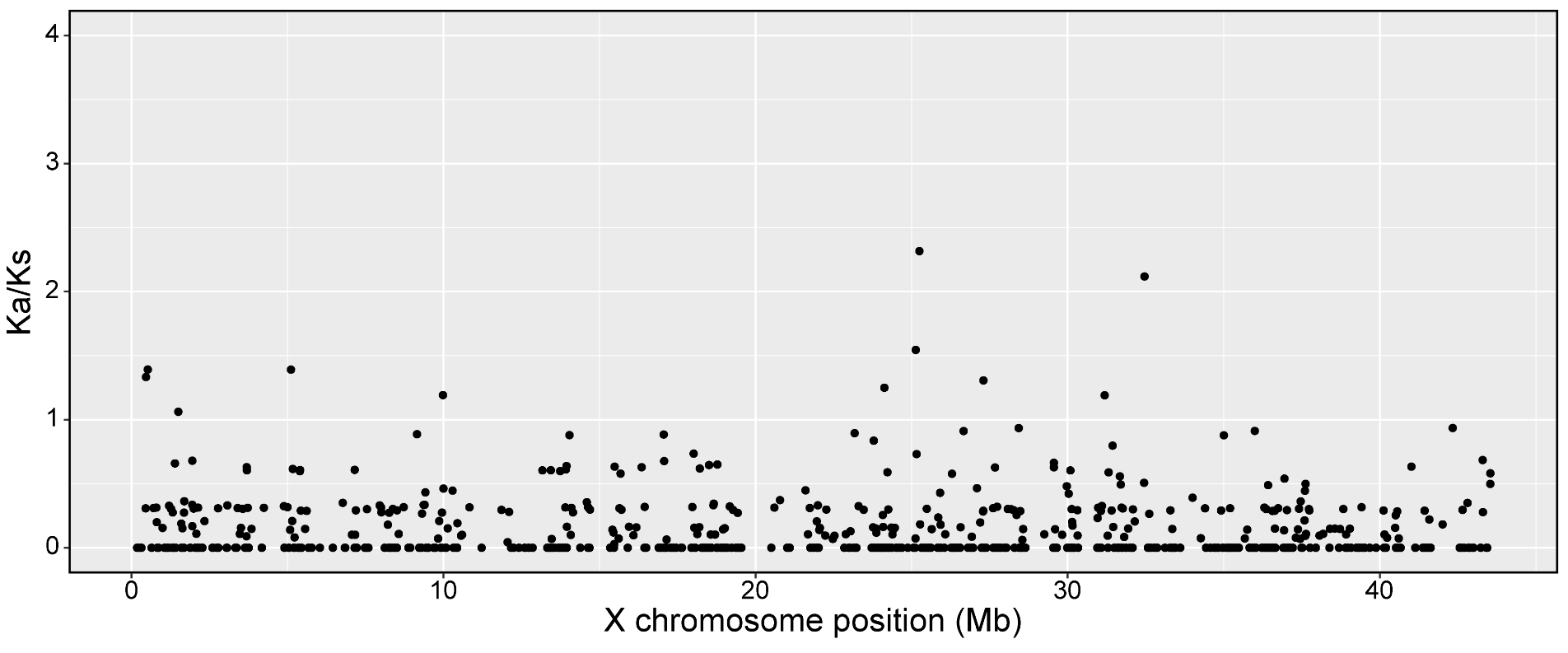


**Supplementary Fig. 7. Ka/Ks between the X and Y chromosomes was estimated for every annotated gene.** Genes are ordered by their position on the X chromosome.

**
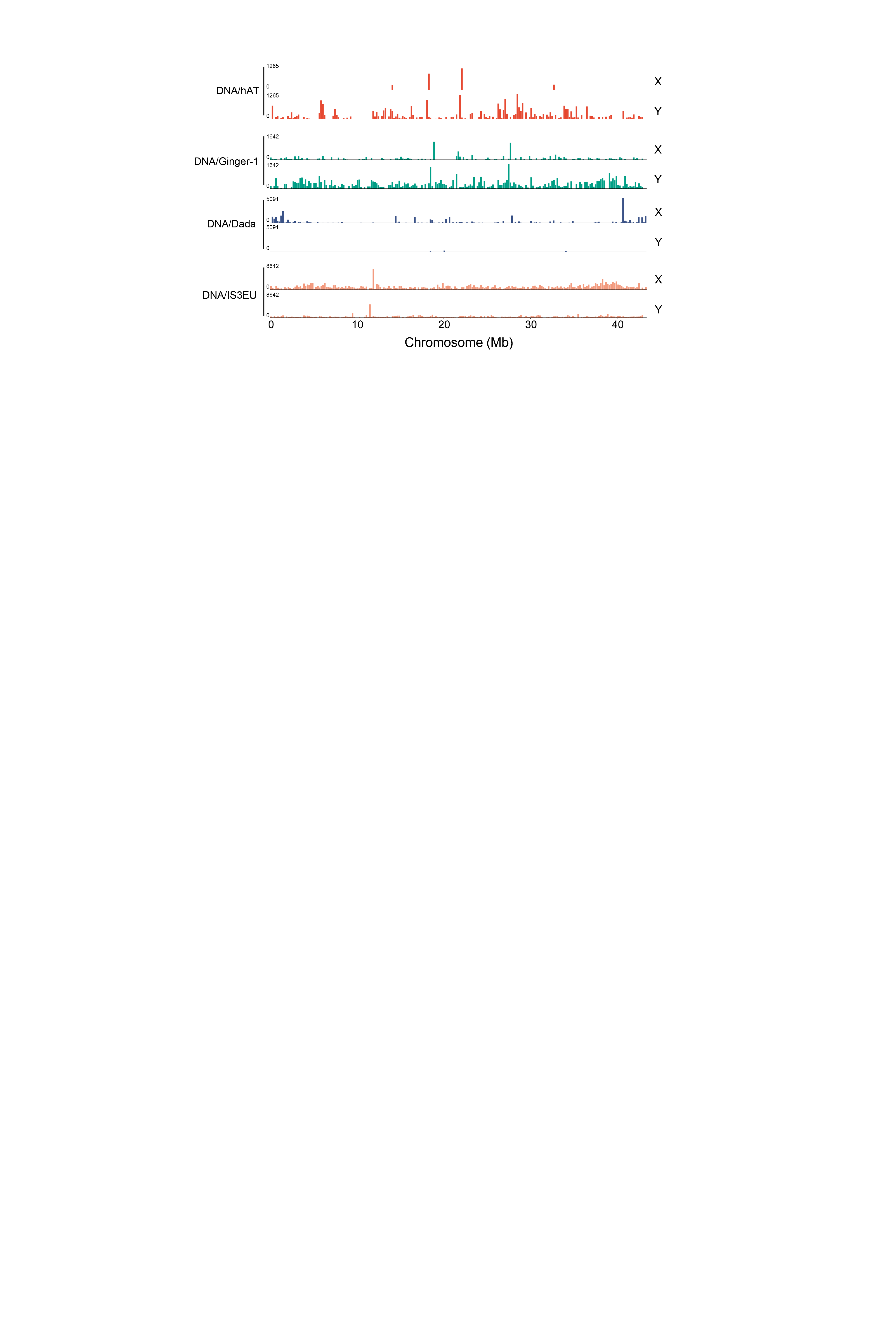
**

**Supplementary Fig. 8. The distribution of DNA/hAT, DNA/Ginger-1, DNA/Dada and DNA/IS3EU sequences on the X/Y chromosomes according to a 200-kb sliding window.**

**
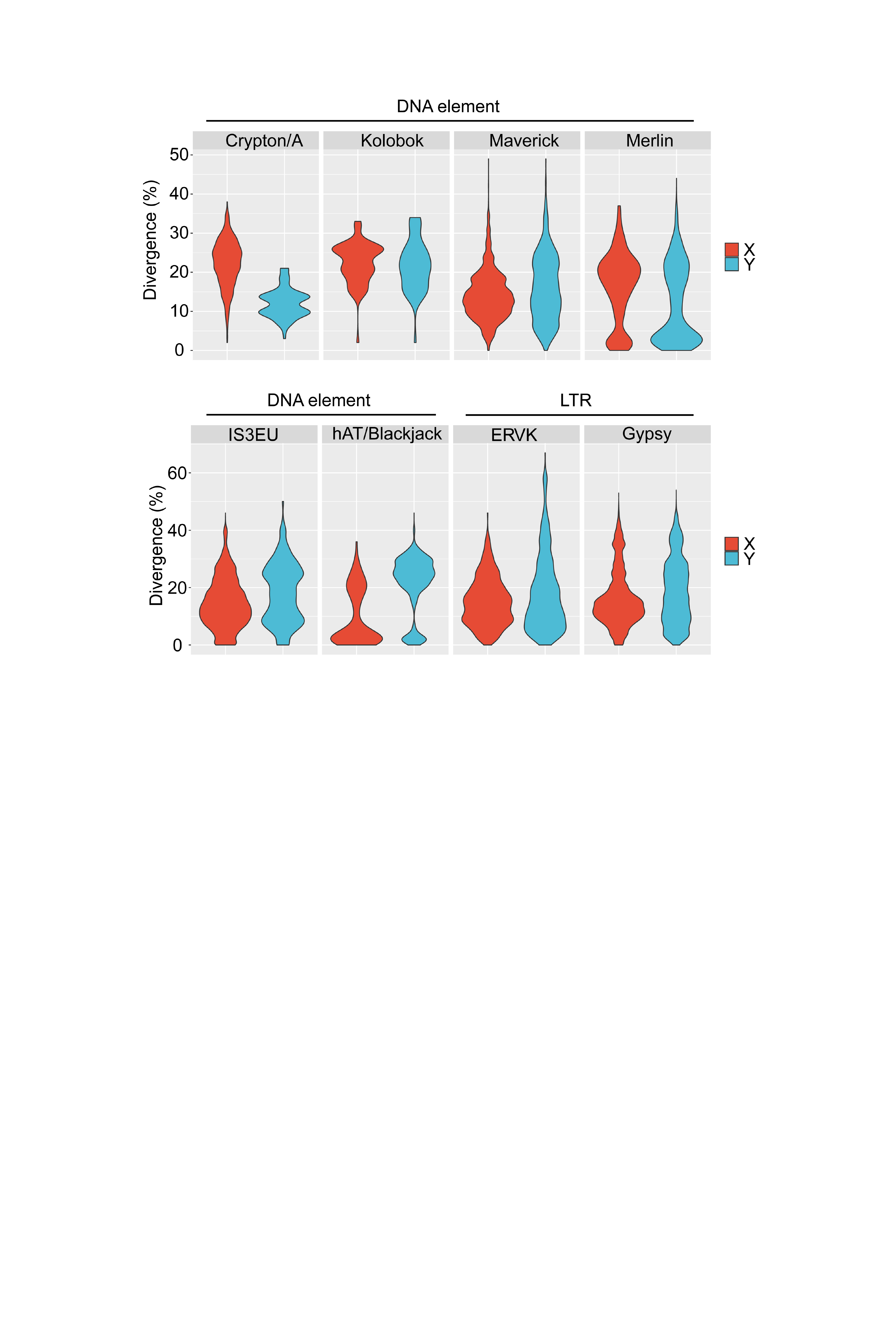
**

**Supplementary Fig. 9. Violin plots showing the frequency distribution of the sequence divergence levels of each transposable element subfamily on sex chromosomes from the inferred ancestral consensus sequences.**


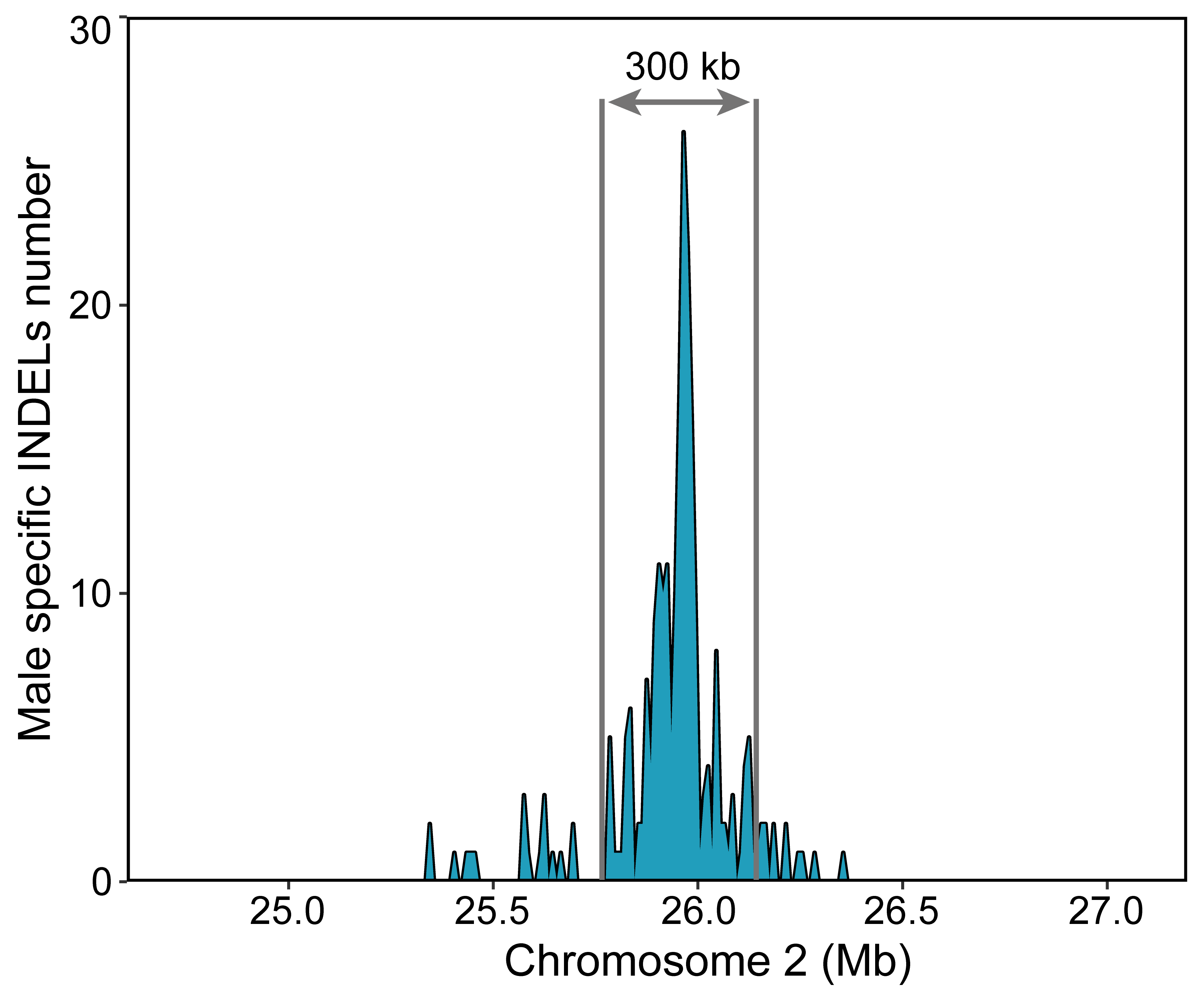


**Supplementary Fig. 10. Identification of an ~300 kb region significantly enriched with male-specific indels on chromosome 2.**

**
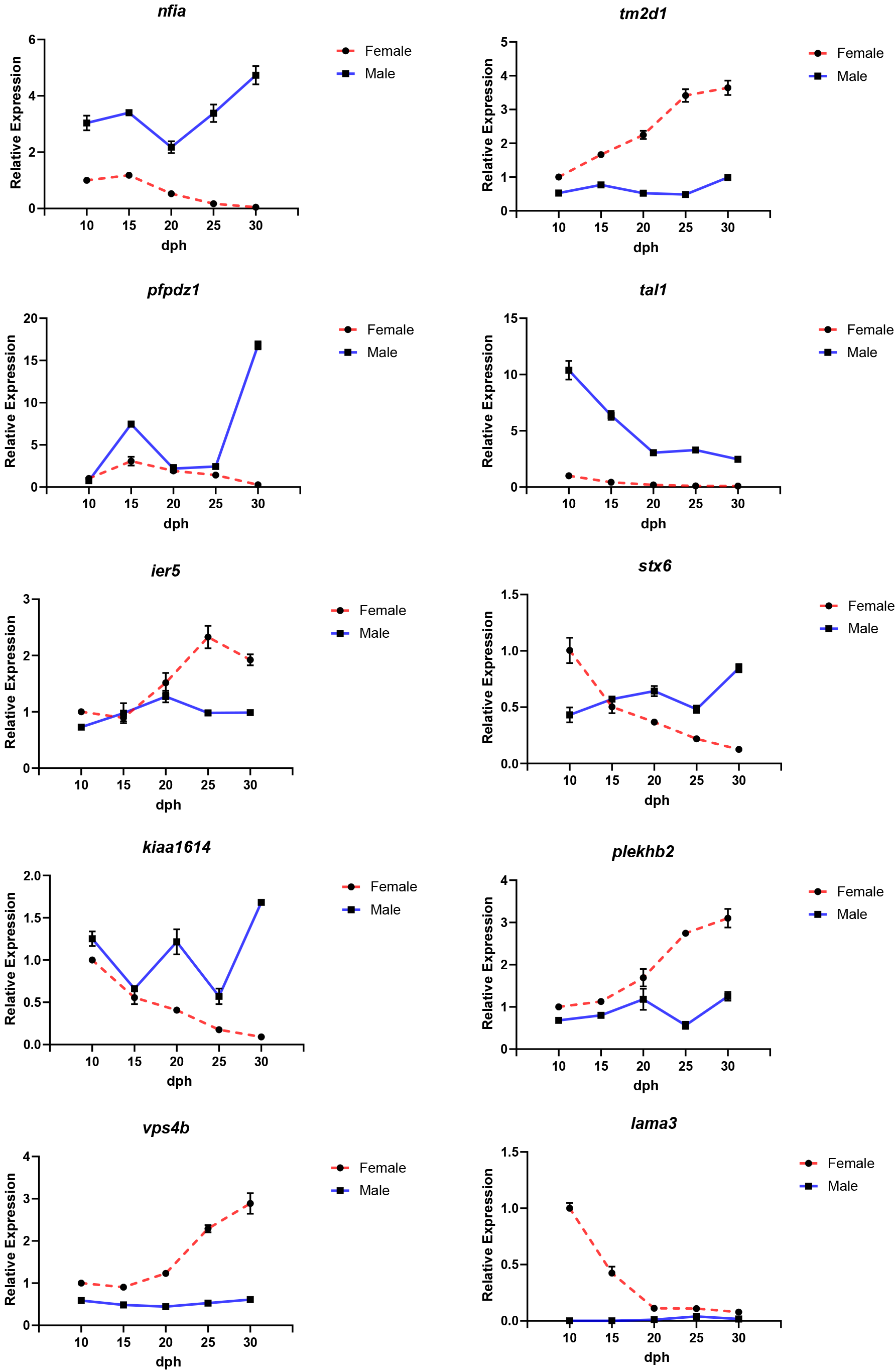
**

**Supplementary Fig. 11. Supplementary Fig. 10. Relative mRNA expression of genes in the SDR during early gonad development.** The cDNAs for qRT-PCR were synthesised with RNAs extracted from gonads of XX and XY fish at 10, 15, 20, 25 and 30 dph (days post-hatching).

**
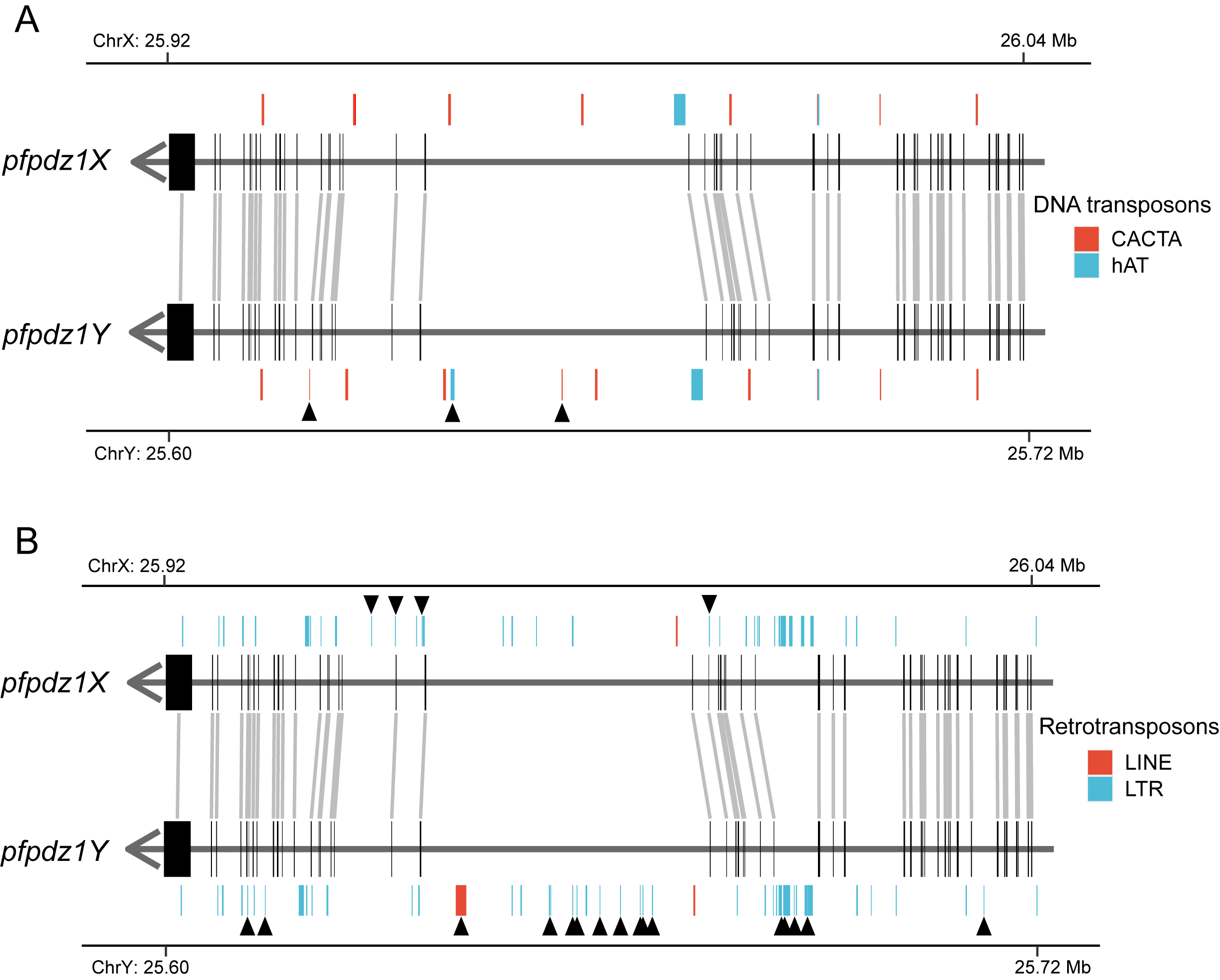
**

**Supplementary Fig. 12. Genomic structure and transposable element distribution of *pfpdz1* on X and Y chromosomes.** The exons and introns are represented by black boxed and dark grey lines between exons, respectively. The links between *pfpdz1X* and *pfpdz1Y* indicate the correspondence between the exons. The *pfpdz1X*- or *pfpdz1Y*-specific transposons are indicated by black triangles. (A) The distribution of DNA transposons on *pfpdz1X* and *pfpdz1Y*. (B) The distribution of retrotransposons on *pfpdz1X* and *pfpdz1Y*.

**
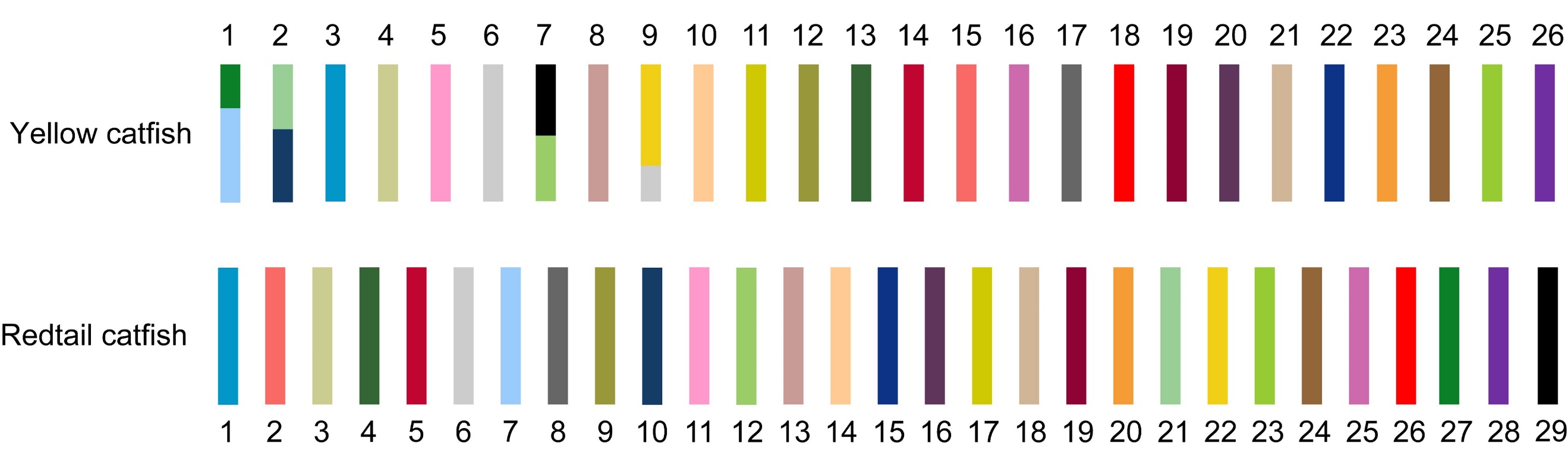
**

**Supplementary Fig. 13. Interchromosomal rearrangements observed between yellow catfish and redtail catfish.** The syntenic blocks are represented by the coloured bar with 29 codes based on their distributions on 29 redtail catfish chromosomes.

**
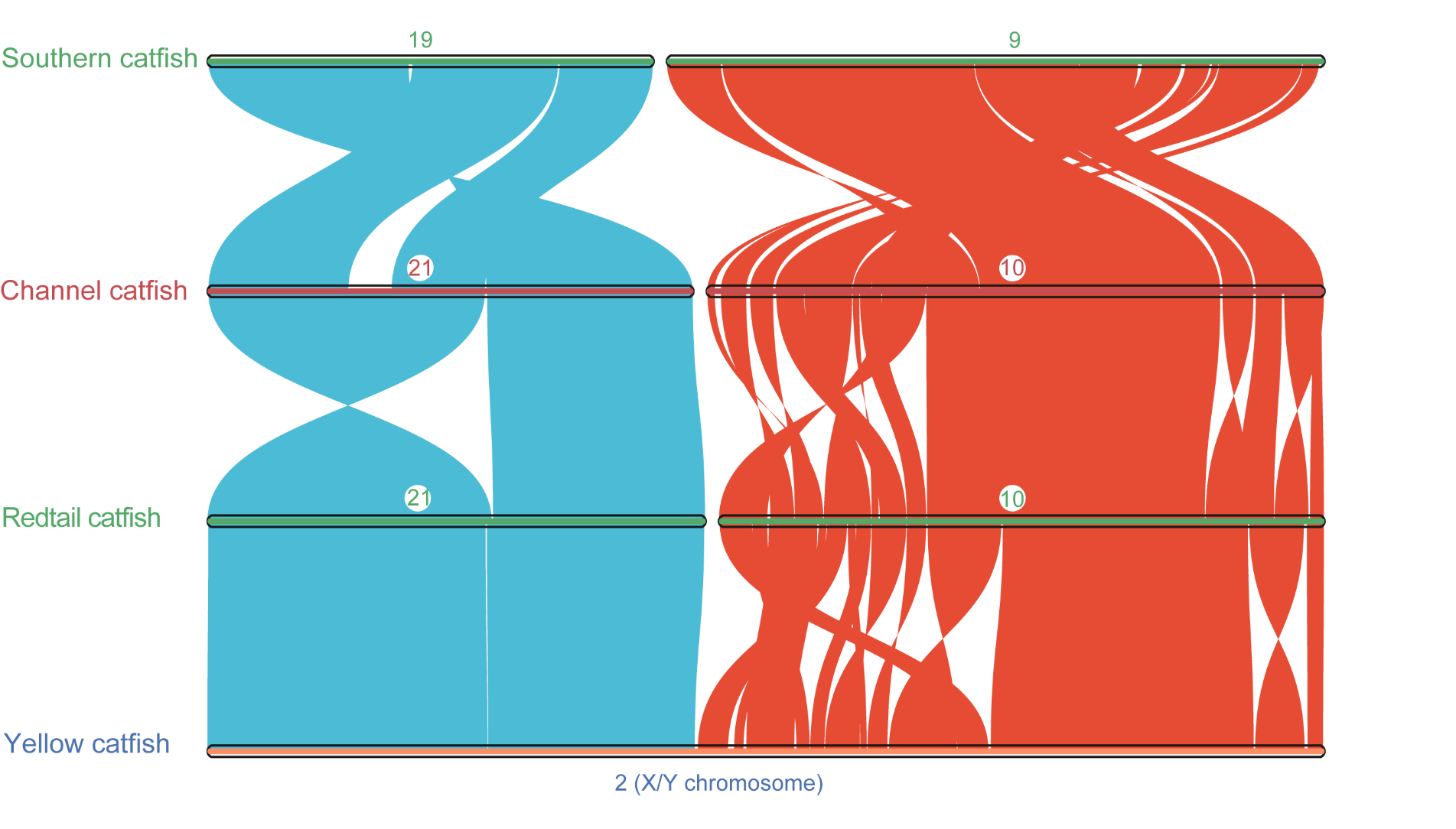
**

**Supplementary Fig. 14. Synteny plot between the sex chromosomes of yellow catfish, Chr21 and 10 of redtail catfish, Chr21 and 10 of channel catfish, and Chr19 and 9 of southern catfish.**

**
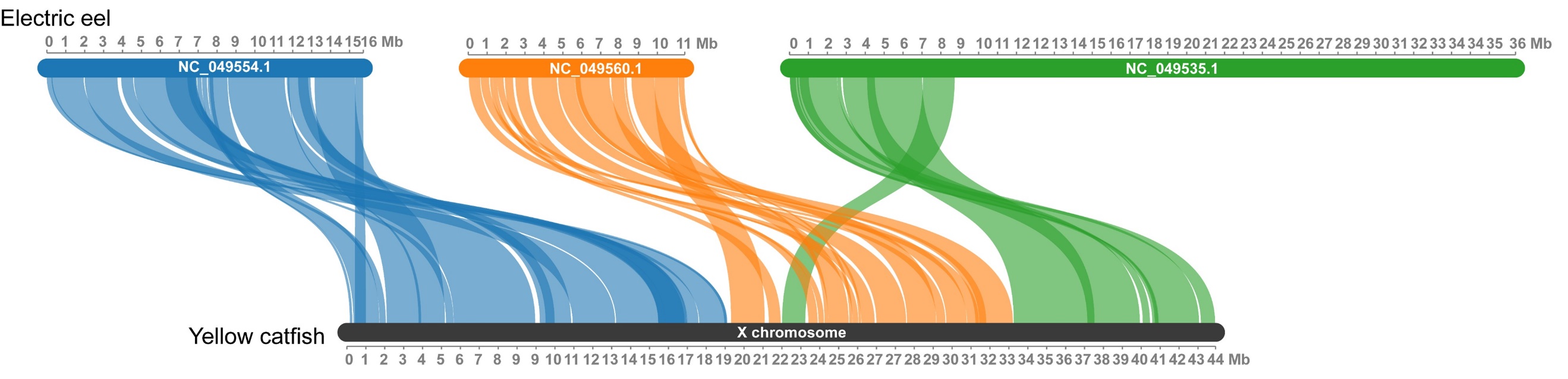
**

**Supplementary Fig. 15. Synteny of the yellow catfish X chromosome and corresponding chromosomes of electric eel based on protein sequences.**

**
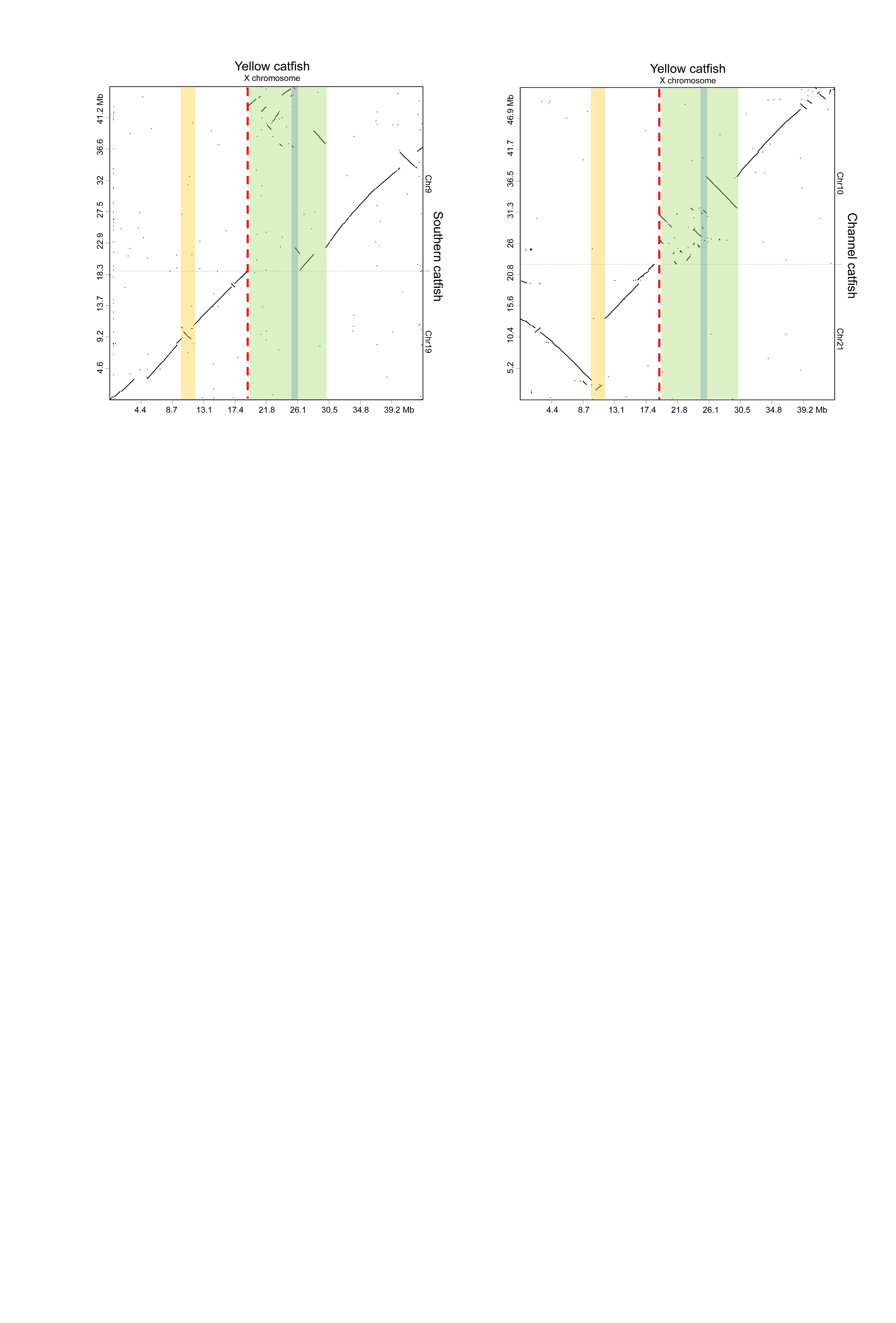
**

**Supplementary Fig. 16. Sequence alignments between the yellow catfish X chromosome and the corresponding chromosomes of southern catfish and channel catfish.** The left panel shows the sequence alignment between the X chromosome of yellow catfish and chromosomes 9 and 19 of southern catfish. The right panel shows the sequence alignment between the X chromosome of yellow catfish and chromosomes 10 and 21 of channel catfish. The putative centromere region is highlighted in yellow, and the highly rearranged region (HRR) is highlighted in green. The SDR is shaded in blue, and the fusion site is divided with a red dotted line.

**
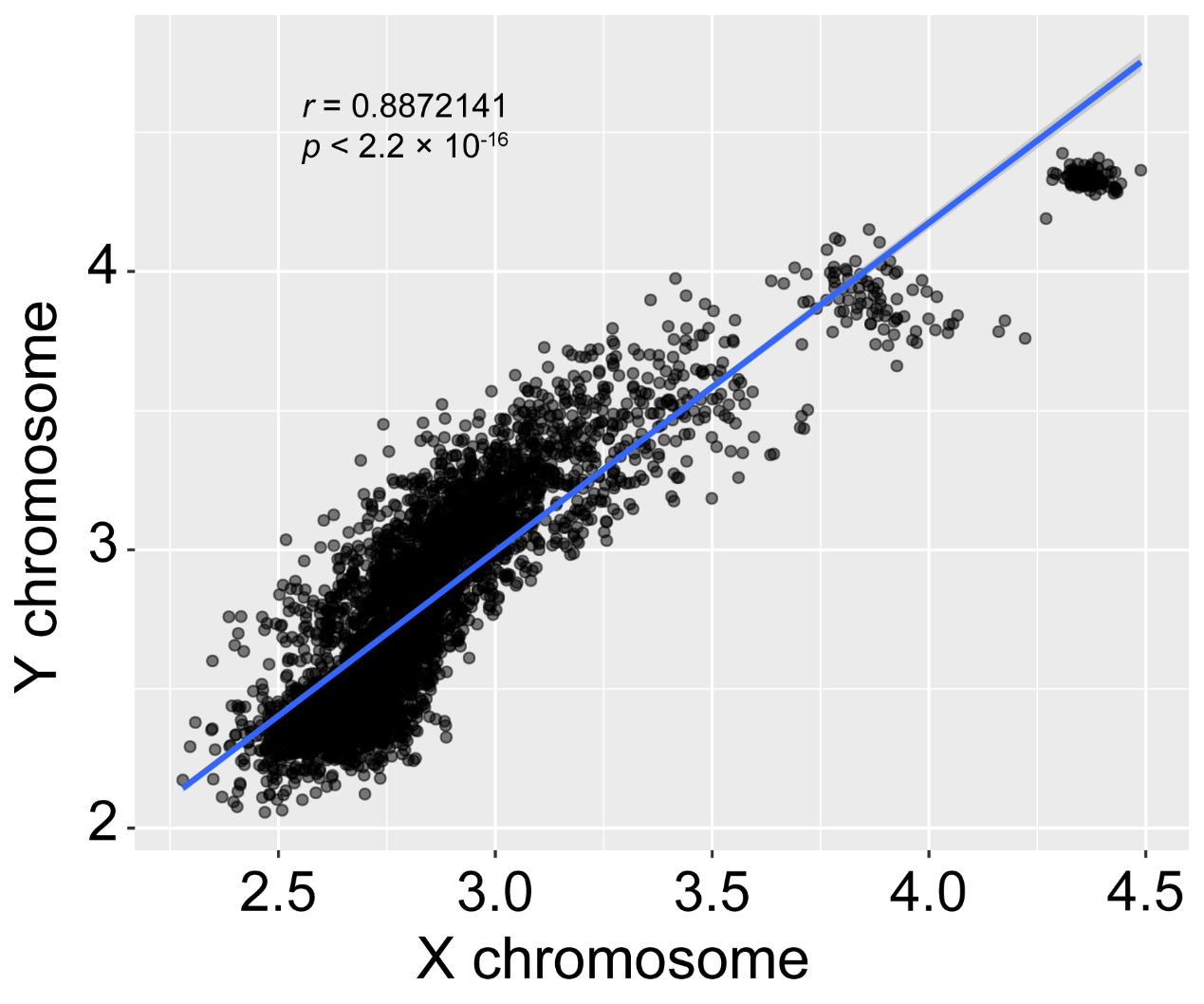
**

**Supplementary Fig. 17. Pearson correlation between the Hi-C contact heatmaps of the X and Y chromosomes.** 100-kb-resolution normalized Hi-C contact heatmaps were used to calculate Pearson correlation coefficients.

**
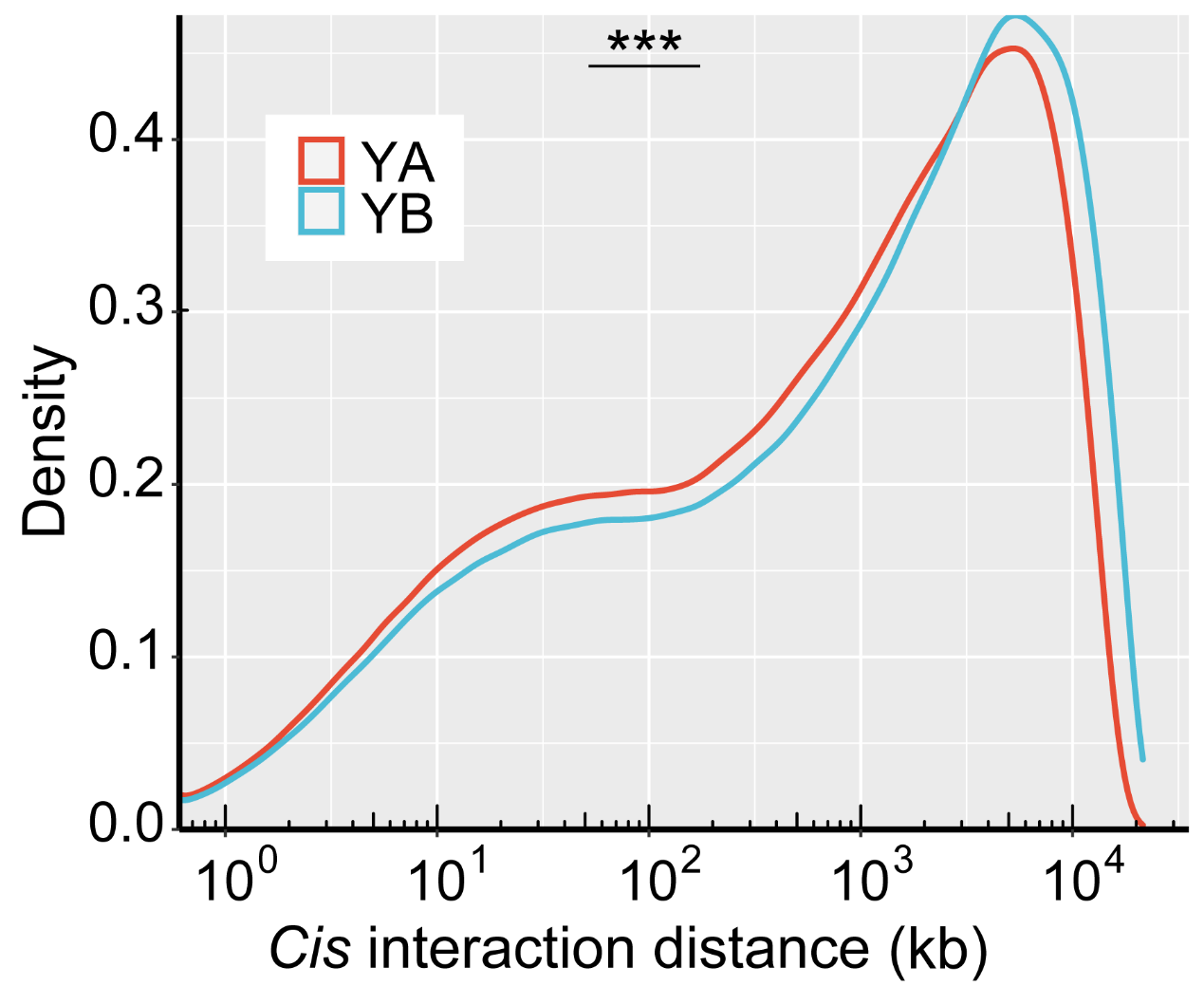
**

**Supplementary Fig. 18.** **Comparison of *cis* interactions between Y1A and Y1B.** All *cis* interactions of pairwise windows within Y1A (ChrY: 0 Mb-17.85 Mb) or Y1B (ChrY: 19.60 Mb-40.65 Mb) were used to plot the density plot. Wilcoxon test, ***: *p* < 2.2×10^-16^.

**
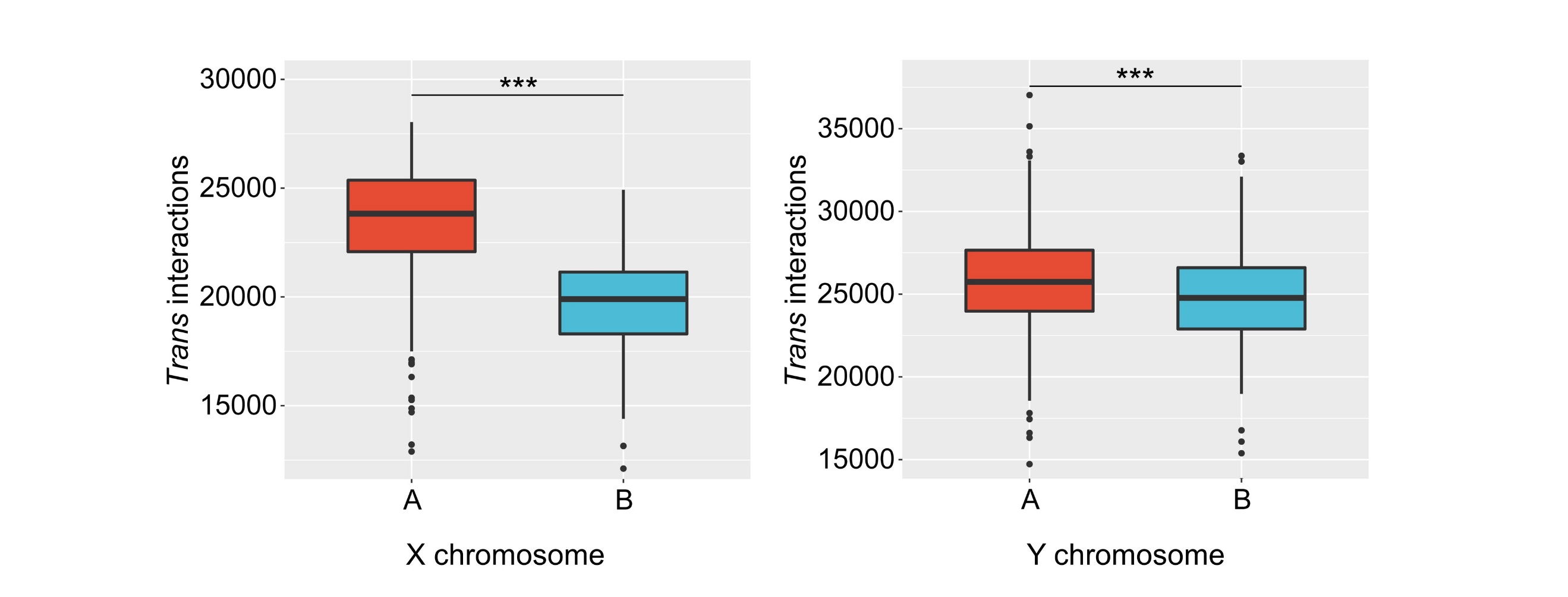
**

**Supplementary Fig. 19. Comparison of *trans* interactions between compartment A/B on sex chromosomes with autosomes.** The *trans* interactions between the X/Y chromosomes and autosomes were counted using a 100-kb window. Wilcoxon test, ***: *p* < 0.001.

**
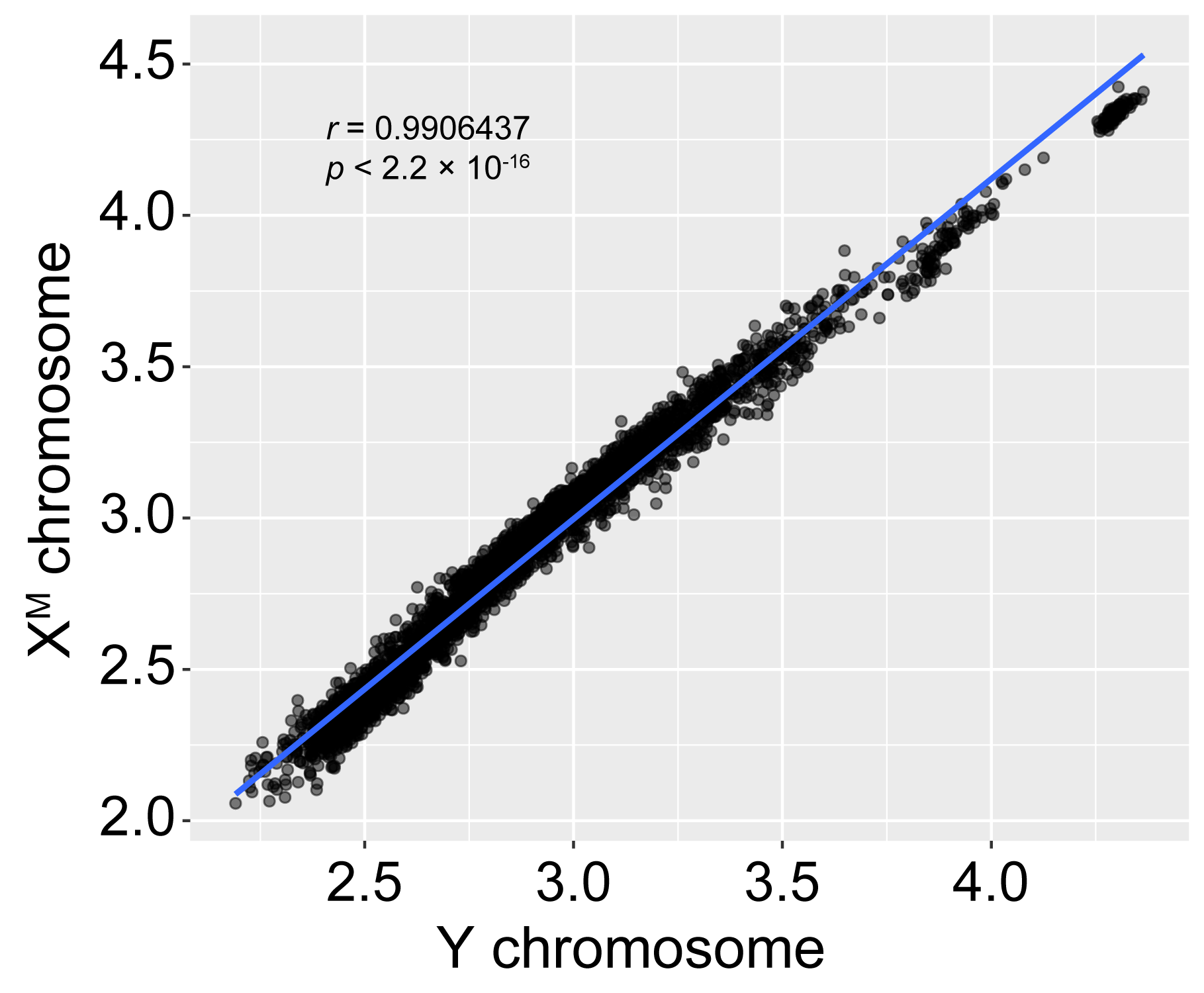
**

**Supplementary Fig. 20. Pearson correlation between the Hi-C contact heatmaps of X^M^ and Y chromosomes.** 100-kb-resolution normalized Hi-C contact heatmaps were used to calculate Pearson correlation coefficients.

**
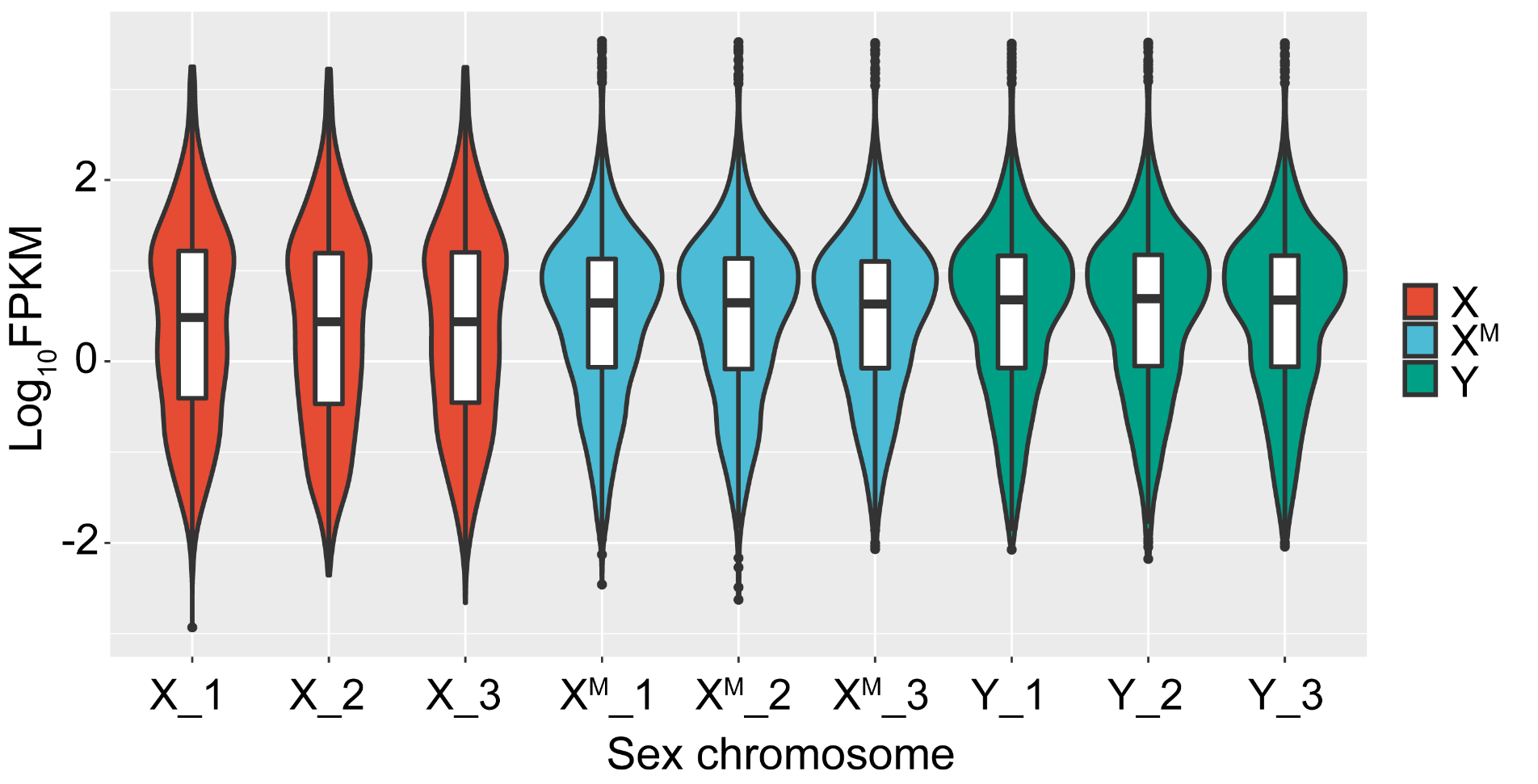
**

**Supplementary Fig. 21. Violin plot showing the expression levels of genes on the X, X^M^, and Y chromosomes.**

**
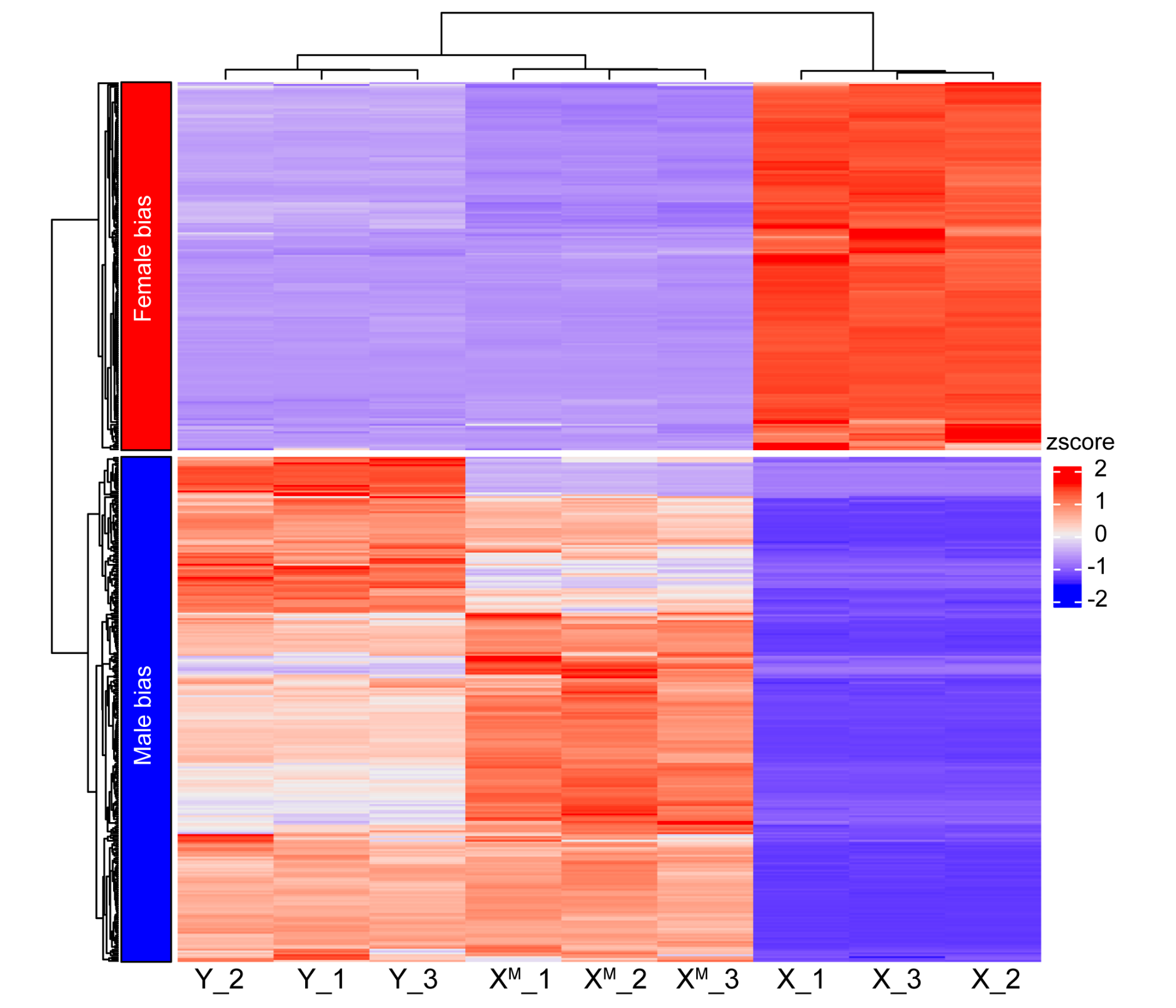
**

**Supplementary Fig. 22. Heatmaps and hierarchical clustering of sex-biased genes on the X, X^M^ and Y chromosomes.** Red represents high expression, and blue represents low expression.

**
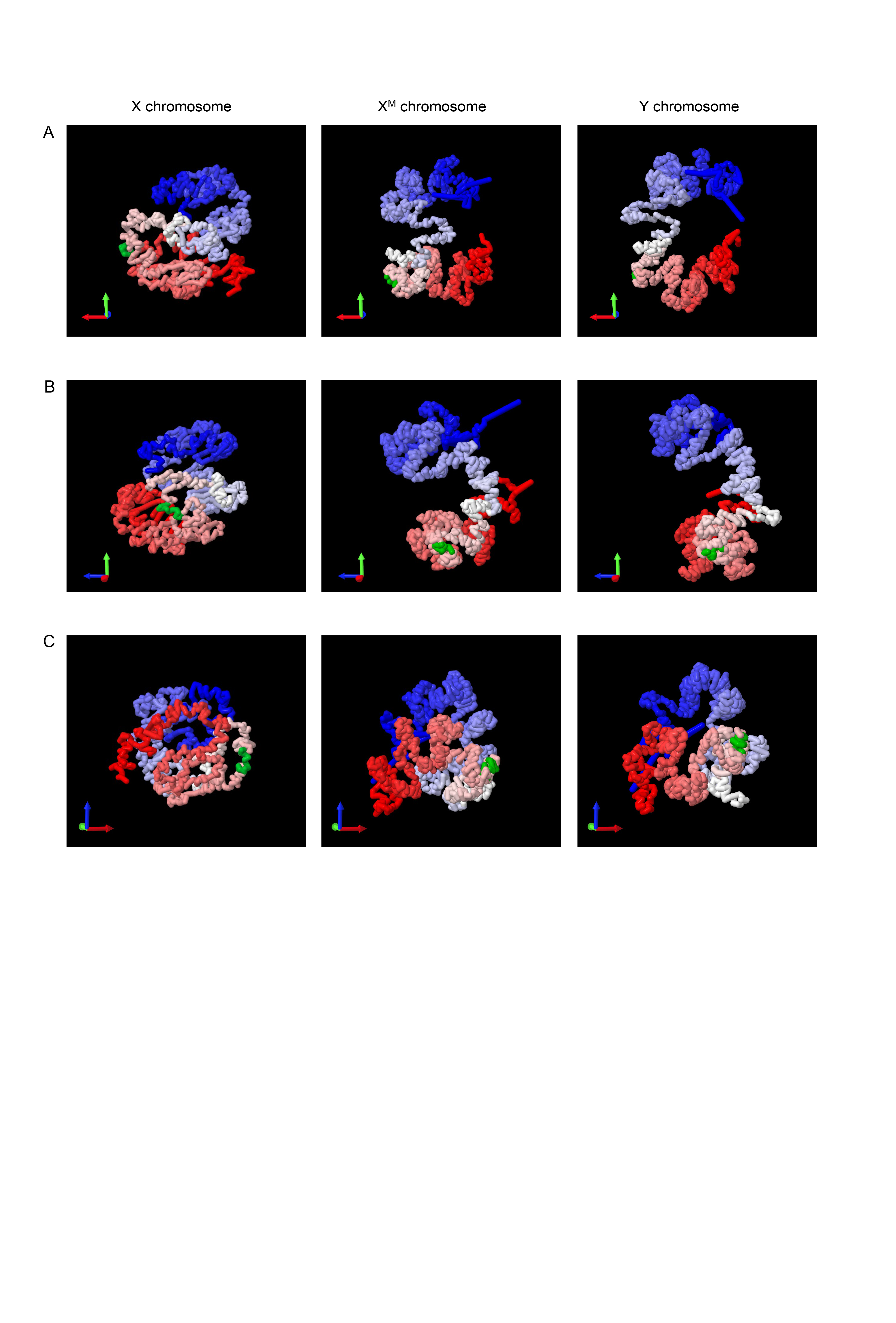
**

**Supplementary Fig. 23. Reconstructed 3D structures of the X, X^M^ and Y chromosomes at different angles.** (A) Dorsal view, (B) right view, and (C) bottom view of 3D structures of the X, X^M^ and Y chromosomes. The SDR is highlighted in green. Blue to red represent the 5' end to 3' ends of the chromosome sequence, respectively.

**
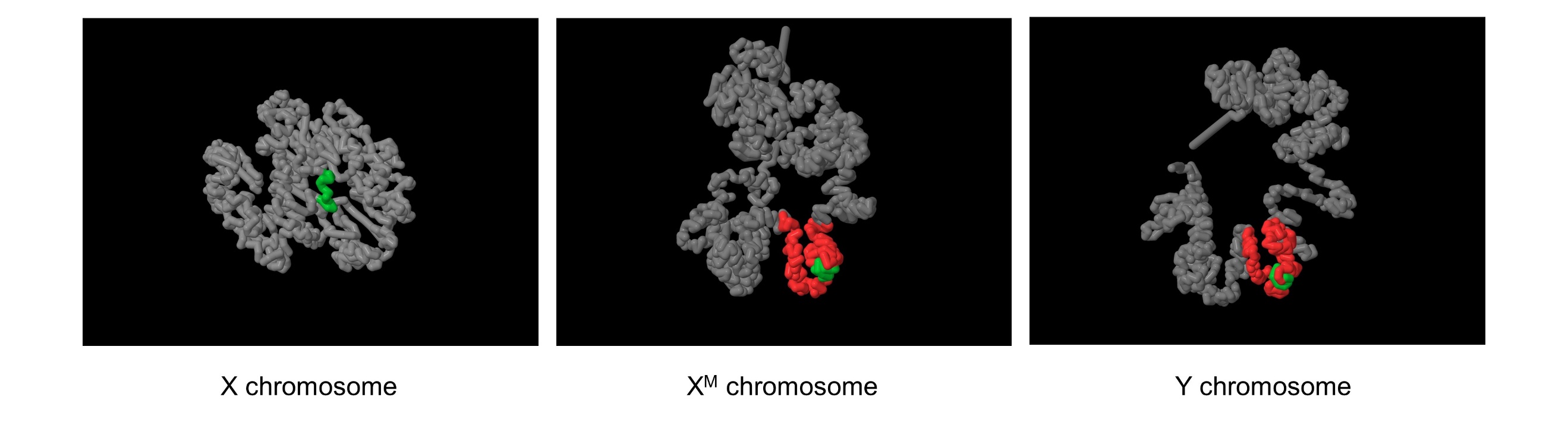
**

**Supplementary Fig. 24. Reconstructed 3D structures of the X, X^M^ and Y chromosomes.** The SDR is highlighted in green. The loop-like regions containing the SDRs of the X^M^ and Y chromosomes are highlighted in red.

**
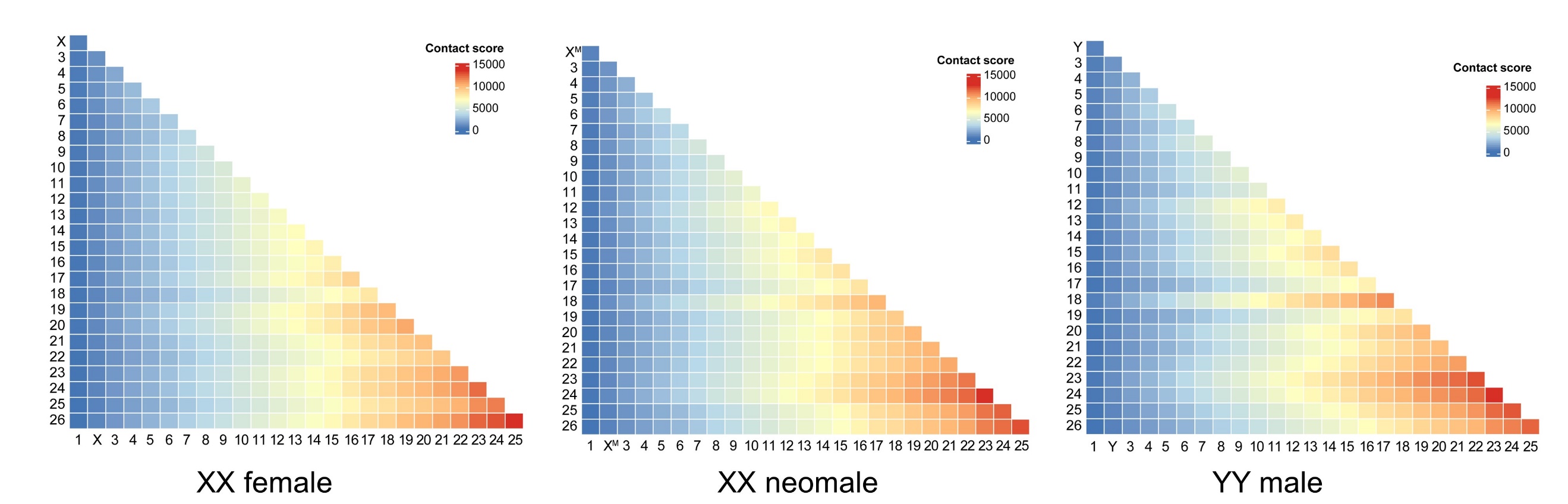
**

**Supplementary Fig. 25. Comparison of relative positions of chromosomes in three kinds of yellow catfish.** Higher scores indicate a shorter distance between two chromosomes.

**
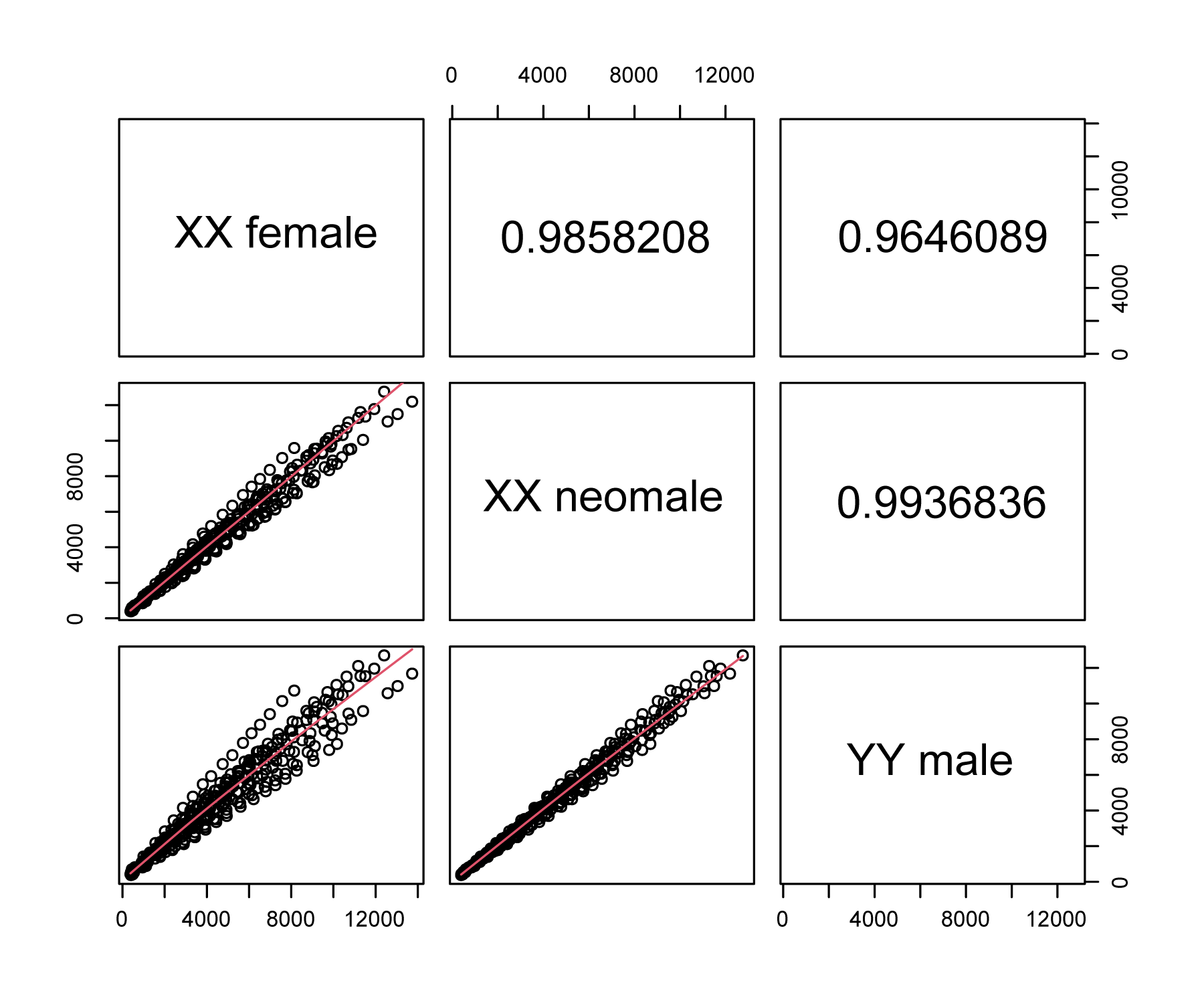
**

**Supplementary Fig. 26. Pearson correlation of chromosome contact scores between XX female, XX neomale and YY male yellow catfish.**

**
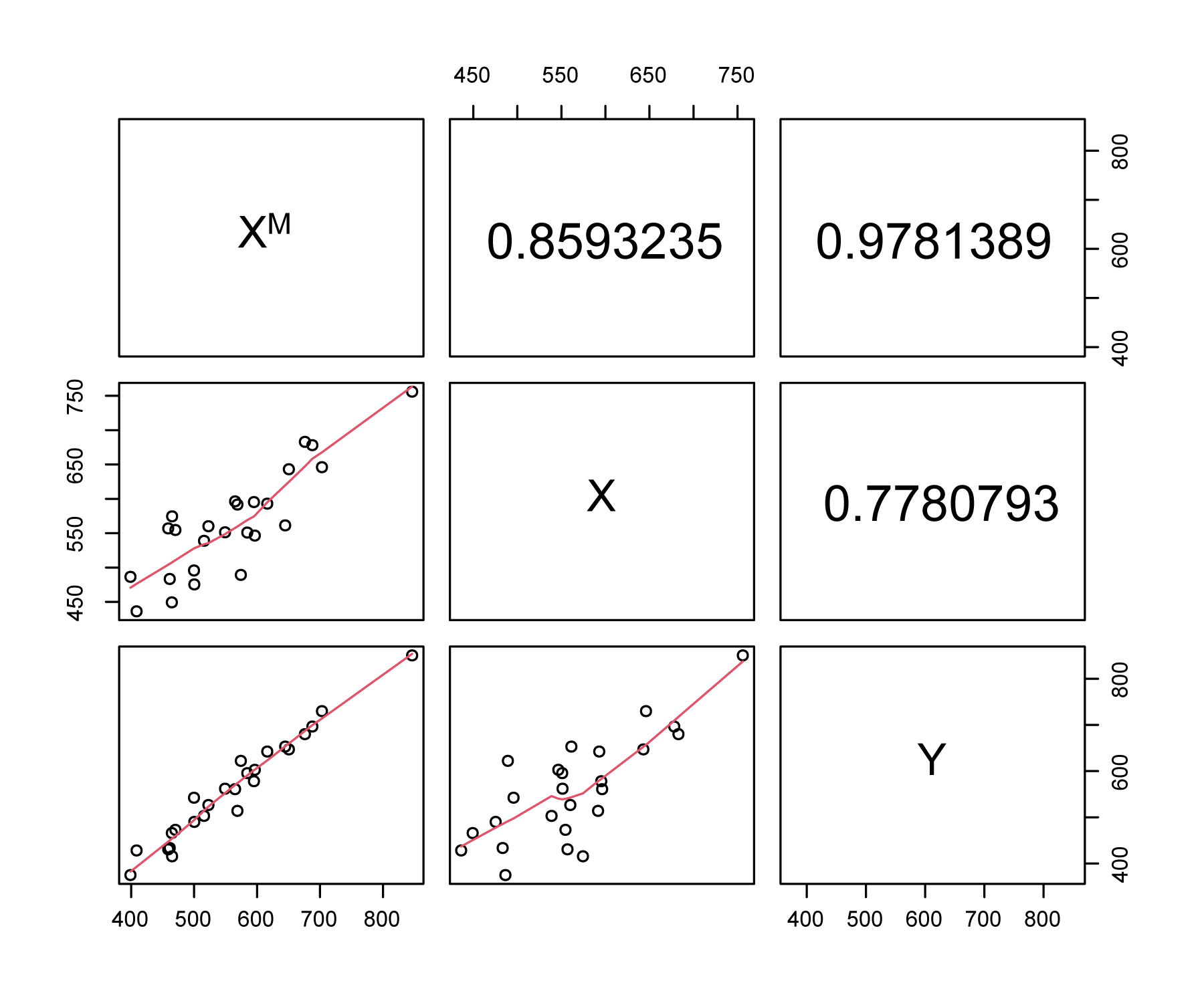
**

**Supplementary Fig. 27. Pearson correlation of chromosome contact scores between the X, X^M^ and Y chromosomes and autosomes.**

**Supplementary tables and table legends**

|  | XX | YY |
| --- | --- | --- |
| Sequence number (contig) | 1335 | 1590 |
| Genome length (bp) | 712,134,760 | 712,074,422 |
| Chromosome length (bp) | 706,994,477  (98.81%) | 703,594,769  (99.29%) |
| Contig N50 (bp) | 3,157,592 | 2,954,525 |
| Scaffold N50 (bp) | 27,162,318 | 27,039,445 |
| BUSCO (%) | 95.0 | 94.7 |

**Supplementary Table 1. Summary of XX and YY genome assemblies­.**

|  | Chromosome X | Chromosome Y |
| --- | --- | --- |
| Sequence number (contig) | 39 | 48 |
| Length (bp) | 43,541,286 | 43,160,729 |
| Longest sequence length (bp) | 9,164,007 | 10,099,044 |
| Contig N50 (bp) | 8,099,354 | 4,357,242 |
| Gene number | 1427 | 1427 |

**Supplementary Table 2. Summary of X and Y chromosome assemblies.**

| Library name | Sample number | | Sex | | Raw data (Gb) | | Clean data (Gb) | | | Mapping rate (%) |
| --- | --- | --- | --- | --- | --- | --- | --- | --- | --- | --- |
| XX pool | | 20 | | XX female | | 19.33 | | 18.72 | 94.50 | |
| YY pool | | 20 | | YY male | | 22.08 | | 21.40 | 94.25 | |
| XX-2 | | 1 | | XX female | | 8.85 | | 7.90 | 98.77 | |
| XX-3 | | 1 | | XX female | | 13.47 | | 12.36 | 98.82 | |
| XX-4 | | 1 | | XX female | | 14.20 | | 12.94 | 98.93 | |
| XX-5 | | 1 | | XX female | | 11.50 | | 10.69 | 98.42 | |
| XX-6 | | 1 | | XX female | | 10.54 | | 9.78 | 98.64 | |
| XX-7 | | 1 | | XX female | | 12.39 | | 11.51 | 98.87 | |
| XX-8 | | 1 | | XX female | | 11.32 | | 9.98 | 98.78 | |
| XX-9 | | 1 | | XX female | | 12.99 | | 12.13 | 98.57 | |
| XX-10 | | 1 | | XX female | | 12.03 | | 11.22 | 98.61 | |
| XX-11 | | 1 | | XX female | | 12.98 | | 12.18 | 98.43 | |
| XX-12 | | 1 | | XX female | | 12.53 | | 11.73 | 98.35 | |
| XX-13 | | 1 | | XX female | | 13.19 | | 12.48 | 98.59 | |
| XX-14 | | 1 | | XX female | | 13.67 | | 12.91 | 98.39 | |
| XX-15 | | 1 | | XX female | | 11.68 | | 11.03 | 98.57 | |
| XX-16 | | 1 | | XX female | | 14.12 | | 13.15 | 98.83 | |
| XX-17 | | 1 | | XX female | | 9.01 | | 8.29 | 98.73 | |
| XX-18 | | 1 | | XX female | | 13.18 | | 12.41 | 98.57 | |
| XX-19 | | 1 | | XX female | | 12.19 | | 11.51 | 98.33 | |
| XX-20 | | 1 | | XX female | | 13.68 | | 12.82 | 98.25 | |
| YY-1 | | 1 | | YY male | | 16.56 | | 15.70 | 98.10 | |
| YY-2 | | 1 | | YY male | | 13.20 | | 12.18 | 98.26 | |
| YY-3 | | 1 | | YY male | | 11.70 | | 11.00 | 98.68 | |
| YY-4 | | 1 | | YY male | | 10.42 | | 9.82 | 98.71 | |
| YY-5 | | 1 | | YY male | | 10.83 | | 10.22 | 98.54 | |
| YY-6 | | 1 | | YY male | | 12.20 | | 11.48 | 98.13 | |
| YY-7 | | 1 | | YY male | | 12.68 | | 12.01 | 98.57 | |
| YY-8 | | 1 | | YY male | | 10.98 | | 10.39 | 98.60 | |
| YY-9 | | 1 | | YY male | | 13.41 | | 12.75 | 98.59 | |
| YY-10 | | 1 | | YY male | | 12.85 | | 12.14 | 98.78 | |
| YY-11 | | 1 | | YY male | | 11.06 | | 10.52 | 98.56 | |
| YY-12 | | 1 | | YY male | | 12.34 | | 11.77 | 98.47 | |
| YY-13 | | 1 | | YY male | | 9.65 | | 9.13 | 98.41 | |
| YY-14 | | 1 | | YY male | | 11.08 | | 10.49 | 98.09 | |
| YY-15 | | 1 | | YY male | | 10.19 | | 9.64 | 98.36 | |
| YY-16 | | 1 | | YY male | | 11.65 | | 11.14 | 98.18 | |
| YY-17 | | 1 | | YY male | | 11.34 | | 10.73 | 98.45 | |
| YY-18 | | 1 | | YY male | | 11.55 | | 11.09 | 98.13 | |
| YY-19 | | 1 | | YY male | | 11.53 | | 10.78 | 99.01 | |

**Supplementary Table 3. Sample information and sequencing read statistics.**

| Gene | SNP count | SNPs in exon region | Start (chr2) | End (chr2) | Length |
| --- | --- | --- | --- | --- | --- |
| *diras1* | 0 | 0 | 25780782 | 25781369 | 587 |
| *lama3* | 5 | 3 | 25797408 | 25821039 | 23631 |
| *vps4b* | 3 | 1 | 25830774 | 25837658 | 6884 |
| *plekhb2* | 1 | 0 | 25839124 | 25844231 | 5107 |
| *kiaa1614* | 17 | 5 | 25849713 | 25865045 | 15332 |
| *stx6* | 30 | 0 | 25870670 | 25882883 | 12213 |
| *ier5* | 0 | 0 | 25886352 | 25888588 | 2236 |
| *tal1* | 33 | 5 | 25907732 | 25915029 | 7297 |
| *pdzk1ip1* | 5 | 0 | 25919931 | 25921994 | 2063 |
| *pfpdz1* | 378 | 18 | 25922712 | 26044137 | 121425 |
| *tm2d1* | 9 | 0 | 26044223 | 26052705 | 8482 |
| *nfia* | 6 | 0 | 26175007 | 26209476 | 34469 |
| *frem1* | 0 | 0 | 26259263 | 26259804 | 541 |
| *atpaf1* | 0 | 0 | 26260574 | 26265661 | 5087 |
| *rab3b* | 0 | 0 | 26266379 | 26273691 | 7312 |
| *nrdc* | 0 | 0 | 26277588 | 26289231 | 11643 |
| *axdnd1* | 0 | 0 | 26291024 | 26298869 | 7845 |

**Supplementary Table 4. Localization of male-specific SNPs in the genes of the SDR.**

**Supplementary Table 5. Gene expression profiles in gonads of XX females, XX neomales and YY supermales (Table S5.csv).**

| Gene symbol | Forward primer (5’-3’) | Reverse primer (5’-3’) |
| --- | --- | --- |
| β-*actin* | CCGTGACATCAAGGAGAAGC | TCGGGACACCTGAACCTCT |
| *nfia* | CAGCCGCATCACATAGGAGT | TTCGGAGACGGTGAAGACG |
| *tm2d1* | AATGCCTTCCTGCACCAAAC | TTCCGATTCCACAGAAACCC |
| *pfpdz1* | AAAATGCCAGAACGTTACTT | TCCTAAGCAGTTAGAGGTCTTC |
| *pdzk1ip1* | ATGGGAAAAGCTGTCCGAG | TCACATGGCTGTCACAGTTTC |
| *tal1* | CTGAGTCCAGCCGCATTC | AAAGGCACCGTTCACATTCT |
| *ier5* | GACTACTACGGCGGAGGATG | TCGTTTCACGGTTCAAAGAG |
| *stx6* | GGAGCAGCAGGATGAGCAGT | CACAGAACAATGAGGAGGATG |
| *kiaa1614* | CATGGTTGTGCCTGACGAGA | TTGCCTTGCGATCTGAGTGA |
| *plekhb2* | CGTTTGTTAAATGCGGATGG | TGCACTGTCTGCACAGAGGC |
| *vps4b* | GGTGCAGATGCAGGGTGTT | TCAGACTGTTGGGCGTGGT |
| *lama3* | AGTTGAGCAAGGCAAAGGG | ACCATTGAGTATGTTGTCCACG |

**Supplementary Table 6. Primers used for qRT-PCR.**

**Reference**

1. Gong G, Dan C, Xiao S*, et al.* Chromosomal-level assembly of yellow catfish genome using third-generation DNA sequencing and Hi-C analysis. *Gigascience*. 2018; **7**: giy120.

2. Kolmogorov M, Yuan J, Lin Y*, et al.* Assembly of long, error-prone reads using repeat graphs. *Nat Biotechnol*. 2019; **37**: 540-6.

3. Ruan J, Li H. Fast and accurate long-read assembly with wtdbg2. *Nat Methods*. 2020; **17**: 155-8.

4. Chin CS, Alexander DH, Marks P*, et al.* Nonhybrid, finished microbial genome assemblies from long-read SMRT sequencing data. *Nat Methods*. 2013; **10**: 563-9.

5. Li H. Aligning sequence reads, clone sequences and assembly contigs with BWA-MEM. *arXiv preprint arXiv:13033997*. 2013.

6. Hu J, Fan J, Sun Z*, et al.* NextPolish: a fast and efficient genome polishing tool for long-read assembly. *Bioinformatics*. 2020; **36**: 2253-5.

7. Dudchenko O, Batra SS, Omer AD*, et al.* De novo assembly of the Aedes aegypti genome using Hi-C yields chromosome-length scaffolds. *Science*. 2017; **356**: 92-5.

8. Durand NC, Shamim MS, Machol I*, et al.* Juicer Provides a One-Click System for Analyzing Loop-Resolution Hi-C Experiments. *Cell Syst*. 2016; **3**: 95-8.

9. Dudchenko O, Shamim MS, Batra SS*, et al.* The Juicebox Assembly Tools module facilitates de novo assembly of mammalian genomes with chromosome-length scaffolds for under $1000. *Biorxiv*. 2018: 254797.

10. Xu M, Guo L, Gu S*, et al.* TGS-GapCloser: A fast and accurate gap closer for large genomes with low coverage of error-prone long reads. *Gigascience*. 2020; **9**: giaa094.

11. Benson G. Tandem repeats finder: a program to analyze DNA sequences. *Nucleic Acids Res*. 1999; **27**: 573-80.

12. Kirov I, Gilyok M, Knyazev A*, et al.* Pilot satellitome analysis of the model plant, Physcomitrellapatens, revealed a transcribed and high-copy IGS related tandem repeat. *Comp Cytogenet*. 2018; **12**: 493-513.

13. Simao FA, Waterhouse RM, Ioannidis P*, et al.* BUSCO: assessing genome assembly and annotation completeness with single-copy orthologs. *Bioinformatics*. 2015; **31**: 3210-2.

14. Chen S, Zhou Y, Chen Y*, et al.* fastp: an ultra-fast all-in-one FASTQ preprocessor. *Bioinformatics*. 2018; **34**: i884-i90.

15. McKenna A, Hanna M, Banks E*, et al.* The Genome Analysis Toolkit: a MapReduce framework for analyzing next-generation DNA sequencing data. *Genome Res*. 2010; **20**: 1297-303.

16. Kofler R, Pandey RV, Schlotterer C. PoPoolation2: identifying differentiation between populations using sequencing of pooled DNA samples (Pool-Seq). *Bioinformatics*. 2011; **27**: 3435-6.

17. Dan C, Lin Q, Gong G*, et al.* A novel PDZ domain-containing gene is essential for male sex differentiation and maintenance in yellow catfish (Pelteobagrus fulvidraco). *Sci Bull*. 2018; **63**: 1420-30.

18. Kent WJ. BLAT--the BLAST-like alignment tool. *Genome Res*. 2002; **12**: 656-64.

19. Kielbasa SM, Wan R, Sato K*, et al.* Adaptive seeds tame genomic sequence comparison. *Genome Res*. 2011; **21**: 487-93.

20. Darling AC, Mau B, Blattner FR*, et al.* Mauve: multiple alignment of conserved genomic sequence with rearrangements. *Genome Res*. 2004; **14**: 1394-403.

21. Tang H, Wang X, Bowers JE*, et al.* Unraveling ancient hexaploidy through multiply-aligned angiosperm gene maps. *Genome Res*. 2008; **18**: 1944-54.

22. Li H. Minimap2: pairwise alignment for nucleotide sequences. *Bioinformatics*. 2018; **34**: 3094-100.

23. Cabanettes F, Klopp C. D-GENIES: dot plot large genomes in an interactive, efficient and simple way. *PeerJ*. 2018; **6**: e4958.

24. Yang Z. PAML 4: phylogenetic analysis by maximum likelihood. *Mol Biol Evol*. 2007; **24**: 1586-91.

25. Adrion JR, Galloway JG, Kern AD. Predicting the Landscape of Recombination Using Deep Learning. *Mol Biol Evol*. 2020; **37**: 1790-808.

26. Rao SS, Huntley MH, Durand NC*, et al.* A 3D map of the human genome at kilobase resolution reveals principles of chromatin looping. *Cell*. 2014; **159**: 1665-80.

27. Servant N, Varoquaux N, Lajoie BR*, et al.* HiC-Pro: an optimized and flexible pipeline for Hi-C data processing. *Genome Biol*. 2015; **16**: 259.

28. Imakaev M, Fudenberg G, McCord RP*, et al.* Iterative correction of Hi-C data reveals hallmarks of chromosome organization. *Nat Methods*. 2012; **9**: 999-1003.

29. Kaul A, Bhattacharyya S, Ay F. Identifying statistically significant chromatin contacts from Hi-C data with FitHiC2. *Nat Protoc*. 2020; **15**: 991-1012.

30. Yu G, Wang LG, He QY. ChIPseeker: an R/Bioconductor package for ChIP peak annotation, comparison and visualization. *Bioinformatics*. 2015; **31**: 2382-3.

31. Xie C, Mao X, Huang J*, et al.* KOBAS 2.0: a web server for annotation and identification of enriched pathways and diseases. *Nucleic Acids Res*. 2011; **39**: W316-22.

32. Zhou Y, Zhou B, Pache L*, et al.* Metascape provides a biologist-oriented resource for the analysis of systems-level datasets. *Nat Commun*. 2019; **10**: 1523.

33. Dixon JR, Selvaraj S, Yue F*, et al.* Topological domains in mammalian genomes identified by analysis of chromatin interactions. *Nature*. 2012; **485**: 376-80.

34. Trieu T, Cheng J. 3D genome structure modeling by Lorentzian objective function. *Nucleic Acids Res*. 2017; **45**: 1049-58.

35. Trieu T, Oluwadare O, Wopata J*, et al.* GenomeFlow: a comprehensive graphical tool for modeling and analyzing 3D genome structure. *Bioinformatics*. 2019; **35**: 1416-8.

36. Dobin A, Davis CA, Schlesinger F*, et al.* STAR: ultrafast universal RNA-seq aligner. *Bioinformatics*. 2013; **29**: 15-21.

37. Li B, Dewey CN. RSEM: accurate transcript quantification from RNA-Seq data with or without a reference genome. *BMC Bioinformatics*. 2011; **12**: 323.

38. Love MI, Huber W, Anders S. Moderated estimation of fold change and dispersion for RNA-seq data with DESeq2. *Genome Biol*. 2014; **15**: 550.

39. Ihaka R, Gentleman R. R: a language for data analysis and graphics. *J Comput Graph Stat*. 1996; **5**: 299-314.
